# Genome-wide investigation of histone acetyltransferase gene family and its responses to biotic and abiotic stress in foxtail millet (*Setaria italica* [L.] P. *Beauv*)

**DOI:** 10.1186/s12870-022-03676-9

**Published:** 2022-06-14

**Authors:** Guofang Xing, Minshan Jin, Ruifang Qu, Jiewei Zhang, Yuanhuai Han, Yanqing Han, Xingchun Wang, Xukai Li, Fangfang Ma, Xiongwei Zhao

**Affiliations:** 1 State Key Laboratory of Sustainable Dryland Agriculture, Shanxi Agricultural University, 030031 Taiyuan, China; 2 College of Agricultural, Shanxi Agricultural University, 030801 Jinzhong, China; 3 Present Address: Shanxi Key Laboratory of Minor Crop Germplasm Innovation and Molecular Breeding, Shanxi Agricultural University, 030031 Taiyuan, China; 4 Beijing Academy of Agriculture and Forestry Sciences, 100097 Beijing, China; 5 Beijing Key Laboratory of Agricultural Genetic Resources and Biotechnology, Institute of Biotechnology, 100097 Beijing, China; 6 College of Life Sciences, Shanxi Agricultural University, Jinzhong, 030801, China

**Keywords:** *SiHAT genes*, Expression analysis, Nitrogen deficiency, Phosphorus deficiency, Abiotic stress, *Sclerospora graminicola* infection

## Abstract

**Background:**

Modification of histone acetylation is a ubiquitous and reversible process in eukaryotes and prokaryotes and plays crucial roles in the regulation of gene expression during plant development and stress responses. Histone acetylation is co-regulated by histone acetyltransferase (HAT) and histone deacetylase (HDAC). HAT plays an essential regulatory role in various growth and development processes by modifying the chromatin structure through interactions with other histone modifications and transcription factors in eukaryotic cells, affecting the transcription of genes. Comprehensive analyses of *HAT* genes have been performed in *Arabidopsis thaliana* and *Oryza sativa*. However, little information is available on the *HAT* genes in foxtail millet (*Setaria italica* [L.] P. *Beauv*).

**Results:**

In this study, 24 *HAT* genes (*SiHAT*s) were identified and divided into four groups with conserved gene structures via motif composition analysis. Phylogenetic analysis of the genes was performed to predict functional similarities between *Arabidopsis thaliana*, *Oryza sativa*, and foxtail millet; 19 and 2 orthologous gene pairs were individually identified. Moreover, all identified *HAT* gene pairs likely underwent purified selection based on their non-synonymous/synonymous nucleotide substitutions. Using published transcriptome data, we found that *SiHAT* genes were preferentially expressed in some tissues and organs. Stress responses were also examined, and data showed that *SiHAT* gene transcription was influenced by drought, salt, low nitrogen, and low phosphorus stress, and that the expression of four *SiHAT*s was altered as a result of infection by *Sclerospora graminicola*.

**Conclusions:**

Results indicated that histone acetylation may play an important role in plant growth and development and stress adaptations. These findings suggest that *SiHATs* play specific roles in the response to abiotic stress and viral infection. This study lays a foundation for further analysis of the biological functions of *SiHATs* in foxtail millet.

**Supplementary Information:**

The online version contains supplementary material available at 10.1186/s12870-022-03676-9.

## Background

Plant nuclear DNA is organized into a DNA-protein structure called chromatin. The central nucleosome consists of 147 bp DNA, usually wrapped on an octamer of histones, comprising two copies each of the core histones H2A, H2B, H3, and H4 [1]. Histone proteins can be extensively modified at their N-terminal tails, which protrude from the core structure of nucleosomes and function as preferred targets for the histone modifiers involved in a series of post-translational modifications (PTMs), including acetylation, methylation, phosphorylation, ubiquitylation, and sumoylation [2, 3]. These modifications of histones play very important roles in gene regulation, genome stability, and genome defense in eukaryotes, mainly by altering the structure of chromatin and/or recruiting regulatory factors [2, 4–6]. Histone acetylation is one of the most studied PTM mechanisms, which alters the physical properties of nucleosomes by weakening interactions between histones and DNA [7]. Histone methylation and other PTMs often create binding sites for other proteins that are bound by specific effector proteins. These can either be involved in the repression of transcription by compacting nucleosome arrays, or they can support transcription by recruiting chromatin remodeling complexes, modifying enzymes, or other complexes involved in elongation or splicing [8–11]. Acetylation of histone proteins at the N-terminus lysine residues plays a crucial role in regulating gene activities in eukaryotes. Acetylation of core histones is correlated with “open” chromatin configurations and is gene activated, whereas deacetylation often produces “closed” chromatin configurations and is associated with gene repression [7, 12, 13]. Similar to most PTMs, histone acetylation is reversible and is dynamically modulated by the action of histone acetyltransferases (HATs) and histone deacetylases (HDACs). Generally, HATs cause gene activation by transferring acetyl groups (CH3COO-) from acetyl-CoA onto the lysine residues of the N-terminal tails of histone proteins [14, 15]. In contrast, HDACs regulate gene repression by removing acetyl groups from these acetylated lysine residues [14, 16]. The targets of HATs and HDACs include H3K9, H3K14, H3K36, H4K5, H4K8, H4K12, and H4K16 [4, 16]. All plant HATs are subdivided into four groups: the general control non-depressible GCN5-related acetyltransferase (GNAT) family; the MOZ, YBF2, SAS3, SAS2, and TIP60 (MYST) family; the camp-responsive element-binding protein (CREB)-binding protein (CBP) family; and the TATA-binding protein-associated factor 1 (TAF_II_ 250) family [14, 17]. Corresponding HATs, which are represented by the acronyms HAG, HAM, HAC, and HAF, respectively [14], play essential roles in regulating gene expression during plant development, exogenous hormone response, and responses to environmental stresses [18–24]. The GNAT group includes three subfamilies: GCN5, elongated complex protein 3 (ELP3), and HAT1-like acetyltransferases, namely HAG1, HAG2, and HAG3 [14]. The GCN5 protein is the catalytic subunit of several multi-protein HAT complexes and plays an essential role in plant development and resistance to abiotic stressors, such as heat, drought, cold, salt, and phosphate starvation [13, 17, 25, 26]. The number of HAT gene family members varies among plants; 12 HATs have been identified in *Arabidopsis* [14], 8 in rice [27], and 32 in tomato [28]. Recently, Kumar et al. [3] provided a comprehensive review of the regulation of histone acetylation during plant growth, development, and stress response.

Foxtail millet (*Setaria italica* [L.] *P. Beauv*), which is one of the most important and ancient cultivated cereal crops, was domesticated in northern China approximately 11,500 years ago [29, 30]. This species exhibits specific morphological features, such as root architectural modifications, small leaf area, thick cell walls, and epidermal cell arrangement, that imparts stress tolerance and high water and nutrient utilization efficiency. These features are bolstered by a small diploid genome (approximately 430 Mb), a short life cycle, and C4 photosynthesis [31, 32]. These characteristics make foxtail millet an ideal model crop for exploring basic biological features, such as plant architecture, physiology, and genome evolution [32, 33]. Additionally, stress tolerance related characteristics of foxtail millet reduce its dependence on synthetic fertilizers, pesticides, herbicides, and insecticides, highlighting it as a model crop for exploring the mechanisms of stress tolerance. With the rapid development of molecular biology, the entire genome of foxtail millet has been sequenced and published by the United States Department of Energy Joint Genomic Institute and Beijing Genomics Institute of China [31, 34], enabling research on the mechanisms of stress response and molecular regulation and providing a foundation for genome-wide analysis of the HAT family members in this species.

In this study, we examined the whole genome of foxtail millet, using bioinformatics analysis, with a focus on the physicochemical properties, chromosomal localization, systematic evolution, gene structure, and conserved domain of the *HAT* gene family. We performed expression profiling in foxtail millet after exposure to abiotic stresses, such as drought, high salinity, low nitrate, and low phosphate, to further identify the function of the *HAT* genes. Additionally, we analyzed the response of *SiHATs* to pathogenic *Sclerospora graminicola* infection. Overall, our results provide a foundation for further study of the functions of *HAT* genes, particularly in the responses to abiotic stresses, and will pave the way for identifying the precise role of *HATs* in plant growth and development.

## Results

### Identification and chromosome mapping of the foxtail millet HAT gene family

**Fig. 1:**
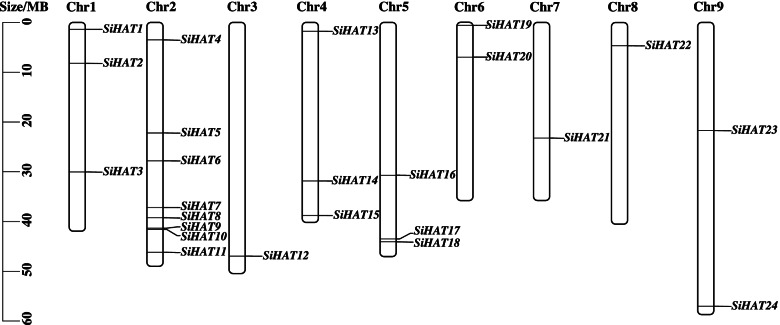
Chromosome locations of histone acetylation genes (*HAT*s) in *Setaria italica*. Chromosomal location was performed on 24 histone acetylation gene family members in *S. italica*

Extensive searches of public and proprietary transcripts and genomic databases, with all previously reported HAT proteins (containing GNAT, MYST, P300/CBP, and TAF1) of rice and *Arabidopsis*, were conducted. A total of 24 HATs were identified in foxtail millet from the Yugu1 genome after excluding redundant genes (Additional file 1). In addition, the position and direction of transcription of each gene were determined on foxtail millet chromosome pseudomolecules available on Phytozome (v12.1) (https://phytozome-next.jgi.doe.gov/), as shown in Fig. 1. The 24 foxtail millet *HAT* genes were found to be distributed on nine chromosomes: eight on chromosome 2; three each on chromosomes 1, 4, and 5; two each on chromosomes 6 and 9; and one each on chromosomes 3, 7, and 8 (Additional file 1, Fig. 1).

Additionally, we analyzed the physical and chemical properties of all HAT family genes and encoded proteins, including the number of amino acids, molecular weight (Mw), isoelectric point (pI), and subcellular location in foxtail millet. The sizes of the 24 predicted SiHAT proteins ranged from 425 aa (SiHAT9) to 5068 aa (SiHAT5), with molecular weights ranging from 38.08 (SiHAT16) to 563.55 kDa (SiHAT5). The pI values ranged from 4.96 (SiHAT20) to 9.86 (SiHAT24). SiHAT21, SiHAT17, and SiHAT5 were determined to be neutral proteins (–0.5 < index of GRAVY values < 0.5), whereas the GRAVY values of the remaining proteins were < 0, indicating hydrophilic properties. Subcellular localization showed that most of the *HAT* genes were located in the nucleus, while two were localized to the mitochondria (*SiHAT6* and *SiHAT24*), and two were cytoplasmic (*SiHAT11* and *SiHAT21*). Only one protein (*SiHAT5*) was localized to the endoplasmic reticulum. More detailed information, including sequence, aliphatic index, instability index, and subcellular localization, are listed in Additional file 1. Prediction of the secondary structure of SiHAT proteins indicated that every member contained α-helix, extended chain, β-folding, and irregular curl structures. The irregular curl and α-spiral structures were the main secondary components, accounting for 30–50% of the secondary structure, while β-folding accounted for only about 5% (Additional file 1).

A chromosome region containing more than two genes within 200 kb is defined as tandem duplication [35]. Homology analysis of *SiHATs* showed that there were two tandem duplication events in the foxtail millet chromosome sequences, each containing *SiHAT9* and *SiHAT10* on chromosome 2 and *SiHAT17* and *SiHAT18* on chromosome 5 (Fig. 1).

### Phylogenetic analysis, motif composition, and structure analysis of SiHATs

**Fig. 2:**
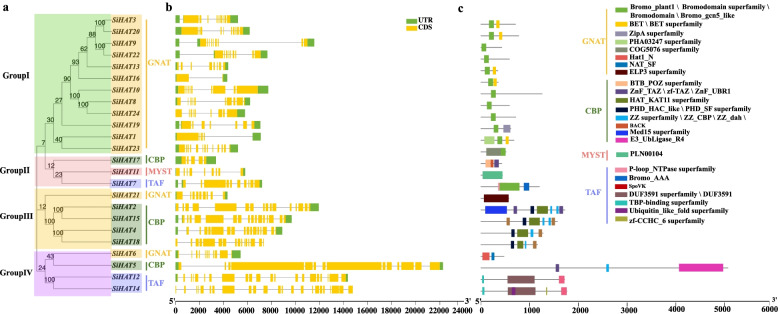
Maximum likelihood phylogenetic trees and structure and conserved domains of histone acetylation gene (*HAT*) in *Setaria italica* (*Si*).** a** Phylogenetic tree and subfamily of *SiHATs*, which are further divided into four groups. **b** The exon-intron organization of *SiHATs.* **c** Conserved domain of the HAT protein in foxtail millet. Bromo domains are conserved domain of GNAT subfamily; PHD, ZnF and ZZ are conserved domains of CBP subfamily, and TBP-binding domains are conserved domain of TAF subfamily

Neighbor-joining phylogenetic analysis was performed, and the 24 SiHAT proteins were clustered into Groups I, II, III, and IV, with 12, 3, 5, and 4 members, respectively (Fig. 2a). Notably, most SiHAT proteins fell into sister pairs (SiHAT3 and SiHAT20, SiHAT9 and SiHAT22, SiHAT12 and SiHAT14), triplets (SiHAT8, SiHAT10, and SiHAT24) or quadruplets (SiHAT2, SiHAT15, SiHAT4, and SiHAT18) in the joint phylogenetic tree (Fig. 2a).

To obtain more insights into gene evolution, the exon-intron organization of *SiHAT* genes was investigated by aligning predicted coding sequences (CDS) against corresponding genomic sequences using the online service Gene Structure Display Server (GSDS). The number of introns in the *SiHAT* family ranged from 2 to 23. Overall, highly similar gene structures and domains were observed for the four HAT subfamilies. In contrast, *SiHAT18* and *SiHAT14* did not contain both upstream and downstream untranslated regulatory regions (UTR), and *SiHAT24* did not contain upstream regulatory regions. The other 21 genes exhibited upstream and downstream regulatory regions (Fig. 2b, Additional file 2). Noticeably, the closest members from the same subgroups had highly similar intron/exon structure (intron number and exon length; Fig. 2b).

To further study the characteristic regions of SiHAT proteins, the motifs of 24 SiHAT proteins were analyzed using Multiple Expectation maximizations for Motif Elicitation (MEME). The results showed that 12 *SiHAT* genes from group I belonged to the GNAT family and possessed the bromodomain. (Fig. 2c). The genes in Group II contained the motifs of the CBP (ZnF_TAZ), MYST(PLN00104), and TAF_1_(Bromo_AAA) family, individually. While the quadruplets genes in Group III belonged to the CBP family and contained the typical motif of the HAT_KAT11 domain, PHD_SF domain, zf-TAZ, and ZZ domains. The five genes in Group IV belonged to the GNAT, CBP, and TAF families. Several HAT proteins possessed unique conserved domains, such as ELP3 in SiHAT21, HAT1 chromodomain, and Znf-C2H2 in the GNAT/MYST family. PHD (Plant Homeodomain), Znf-ZZ, and Znf-TAZ domains were observed in the CBP family. These four subfamily proteins were also compared with other typical GNAT/CBP/TAF/MYST conserved domains in *Arabidopsis* and *O. sativa* (Additional files 3, 4, 5 and 6). These conserved domains allowed SiHATs to interact with RNA Pol II during transcript elongation, bind with the transactivation domain of transcription factors and acetylated histone lysine residues, and interact with co-factors (Additional file 2). Interestingly, the sister pair genes also had the same structure and conserved domain, indicating they may have the same function in foxtail millet.

### Phylogenetic relationship and collinearity analysis of HATs in *Setaria italica*, *Oryza sativa*, and *Arabidopsis thaliana*

**Fig. 3:**
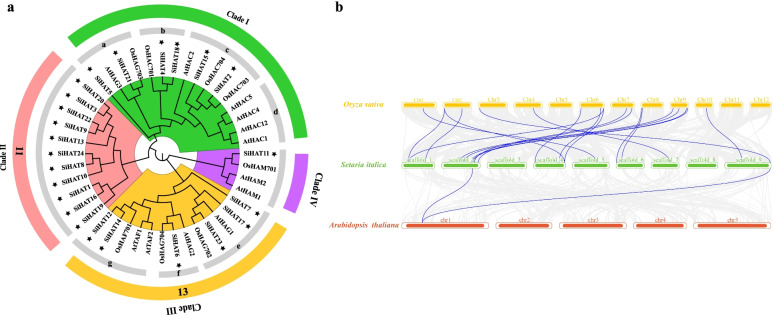
Phylogenetic analysis and Collinearity analysis of histone acetylation proteins (HATs) in *Arabidopsis thaliana (At), Oryza sativa (Os)*, and *Setaria italica (Si)*. **a** Phylogenetic tree of *HAT* genes. **b** Collinearity of foxtail millet *HAT* and related species. The green rectangular color block represents the foxtail millet chromosome, yellow rectangular color block represents the rice chromosome and red rectangular block represents Arabidopsis chromosome. The number represents the chromosome number

To better understand the phylogeny of the foxtail millet *HAT* gene family, the *SiHAT*s were subjected to synteny analysis with *HAT* genes of the typical model plants: the dicot *Arabidopsis thaliana* and monocot *Oryza sativa.* A total of 19 *SiHAT* genes were synchronized with those in *O. sativa*, thus, a phylogenetic tree was constructed using the protein sequences of all 24 *SiHAT*s, 19 *OsHAT*s, and 12 *AtHAT*s. These 55 HAT proteins were divided into four clades (Fig. 3a). The results showed the phylogenetic relationship of HAT proteins between dicots and monocots. Apart from Clade II, which is the unique group of foxtail millet, containing 11 *HAT* gene family members, the other clades included HAT proteins from the three species, suggesting that these genes existed before the divergence of monocots and dicots. Clade I was further subdivided into 4 classes, namely a, b, c, and d, with 4, 3, 5, and 4 members respectively. Clade III was further divided into classes e, f, and g (Fig. 3a). Further, we analyzed the 11 SiHATs in Clade II and found they all belonged to the GNAT family, possessed a Bromo domain, and had structures obviously different from those of the other family members in other clades (Additional files 3 and 7).

The ratio of non-synonymous to synonymous (*Ka/Ks*) nucleotide substitutions was calculated to investigate the selection pressure on *SiHAT*s [36]. The *SiHAT* genes of foxtail millet were subjected to one-to-one orthologous analysis with its homologous genes in *Arabidopsis thaliana* and *Oryza sativa*. (Fig. 3b, Additional file 8). In total, 19 *SiHAT* genes displayed a syntenic relationship with those in *Oryza sativa*, while there were two homologous pairings in *Arabidopsis thaliana*. The results indicated that the foxtail millet and rice *HAT* genes were genetically similar. We also found that some *SiHAT* genes were evolved from rice and *Arabidopsis*, respectively (Fig. 3b, Additional file 8).

### Cis-elements analysis of *SiHAT* promoters

**Fig. 4:**
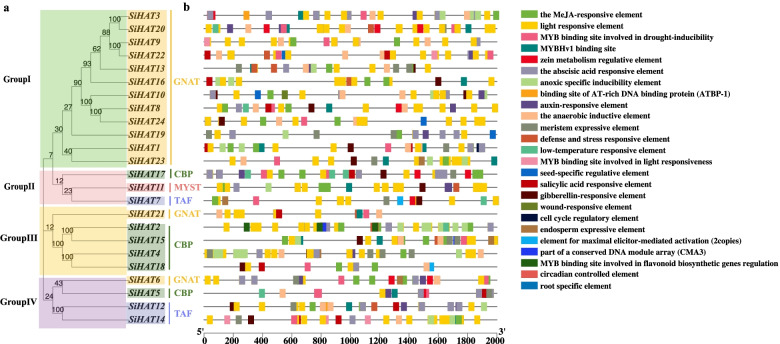
Maximum likelihood phylogenetic trees and prediction of cis-acting elements in promoter of histone acetylation genes ( *HAT* s) in *Setaria italica (Si)*. **a** Phylogenetic trees of foxtail millet *HAT* genes. **b** Promoter analysis of foxtail millet HAT genes. The 2 Kb promoter sequences of corresponding HAT genes were used to analyze hormone-related *cis*-elements, plant growth and development *cis*-elements and stress-related elements. Different *cis*-elements were indicated by different colored symbols and placed in their relative position on the promoter of *SiHATs*

To further investigate the putative functions of *SiHAT* genes, a plant promoter database (PlantCARE) search was conducted in the promoter regions at 2000-bp upstream of the transcription initiation site of *SiHAT* genes. As shown in Fig. 4 and Additional file 9, three main categories of *cis* elements were found in the promoter sequences of *SiHAT* genes. The first category was involved in phytohormones, such as abscisic acid (ABA), methyl jasmonate (MeJA), auxin, and salicylic acid (SA). The second category was associated with stresses, such as anaerobic induction, drought inducibility, low-temperature responsiveness, pathogen infection, wound responsiveness, and salt inducibility. The last category was related to plant growth and development, such as zein metabolism regulation. Meristem, root, endosperm inducibility (GC-motif), and abscisic acid responsive elements were found in almost all gene promoters. Importantly, all the 24 *SiHAT* genes contained the light responsive element, while the MeJA-responsive element (TGACG-motif and CGTCA-motif), anoxic specific inducibility element (GC-motif), and the abscisic acid responsive element (ABRE) were found in almost all gene promoters. Interestingly, distinct differences in *cis* elements between the sister pair genes, including the GA and MeJA response elements, were found in the promoter of *SiHAT3*, whereas ABA and defense and stress response elements were found in *SiHAT20*. These results showed that *SiHAT*s may have affected hormone signal responsiveness, stress adaptation, and development. No cytokinin-responsive elements were identified in these promoter regions.

### Spatial and temporal expression of *SiHAT* genes

**Fig. 5:**
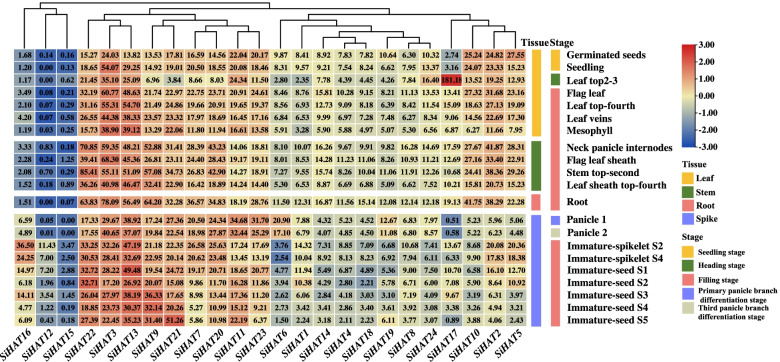
Relative expression patterns of histone acetylation genes (*HAT*s) in different tissues of *Setaria italica*. Heat maps reflect the fragments per kilobase of transcript per million mapped fragments (FPKM) of *HAT*s. Color from red to blue indicates high to low expression

To obtain insight into the expression patterns of *SiHAT* genes in various tissues, a heat map was generated using the gene expression data in the foxtail millet Exp database. The results showed complex specific and overlapping *SiHAT* expression in various tissues and organs. The expression level of the same gene varied among tissues and organs; for example, *SiHAT17* was highly expressed in top leaves 2–3 days after the heading stage. In contrast, low or no expression signals were detected in panicles. On the other hand, the expressions of different genes were also notably different in the same tissues and organs. For example, in the root at the filling stage, the expression of *SiHAT3, SiHAT9, SiHAT13*, and *SiHAT22* from the *GNAT* gene family was significantly higher than that of other genes. Some genes were exclusively expressed in single tissues or organs; for example, *SiHAT17* was expressed in leaf top after 2–3 days, and *SiHAT16* expression was observed in immature ears. In addition, *SiHAT3*, *SiHAT13*, and *SiHAT22* were highly expressed in all tissues at different developmental stages, whereas two *SiHAT15* and *SiHAT12* exhibited almost no expression in any of the tested tissues (Fig. 5). These results demonstrated that the expression patterns of *SiHAT*s differed among tissues and were associated with plant growth and development.

### Expression analysis of *SiHAT*s under stress

To confirm whether the expression of *SiHAT* genes could be regulated by abiotic and biotic stress, we tested the effects of abiotic stresses such as nitrate deficiency and phosphate deficiency, salt-alkali, and drought.

**Fig. 6:**
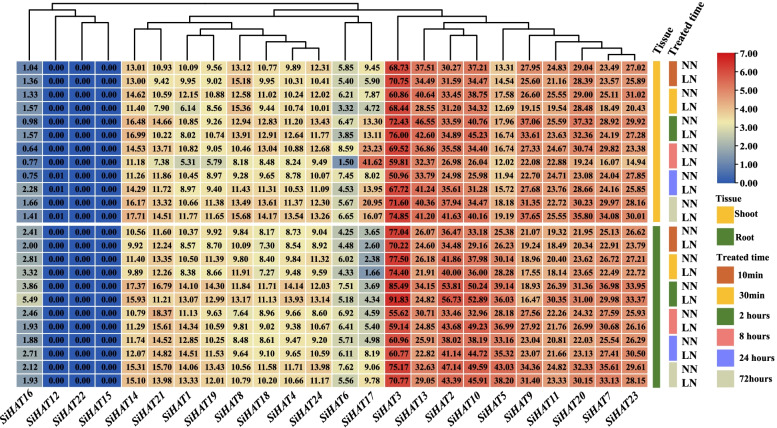
Expression analysis of histone acetylation genes (*HAT*s) in *Setaria italica* under low nitrogen stress. Low nitrate stress time represented by 10 min, 30 min, 2, 8, 24, and 72 h, NN and LN represent normal and low nitrogen treatment, respectively. Leaf and Root describe tissues sampled

Under low nitrate conditions, the expressions of most *SiHAT* genes were either slightly upregulated or downregulated. Most genes were downregulated after 2 h, which was the inverse of what was seen at 24 h. The expression of several genes (*SiHAT17*, *SiHAT8*, and *SiHAT5*) were clearly different between shoots and roots. *SiHAT3* expression was continuously upregulated under low nitrate conditions in the shoot, but was only upregulated at 2 h in the root (Fig. 6). This suggests that *SiHAT3* likely performs different control functions during nitrate absorption and transport.

**Fig. 7:**
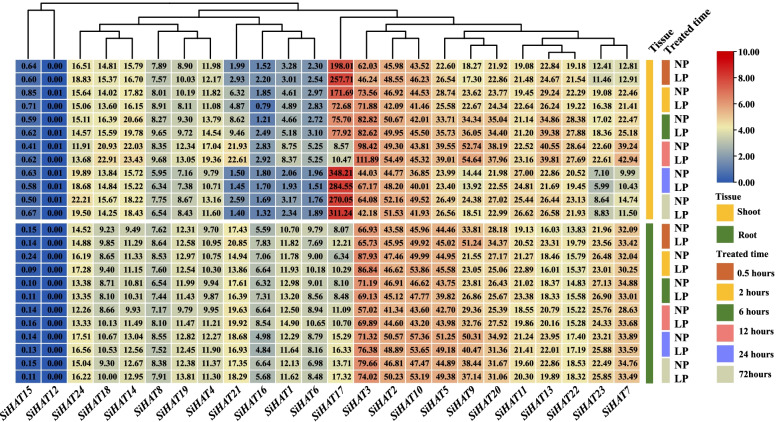
Expression analysis of histone acetylation genes (*HAT*s) in *Setaria italica* under low phosphorus stress. Low phosphorus stress time represented by 0.5, 2, 6, 12, 24, and 72 h. NP and LP represent normal and low phosphorus treatments, respectively. Leaf and Root describe tissues sampled

Most *SiHAT* genes were not strongly upregulated under low phosphate conditions, while five *SiHAT* genes (*SiHAT1*, *SiHAT6*, *SiHAT7*, *SiHAT19*, and *SiHAT21*) were strongly upregulated in roots and weakly upregulated in shoots. In contrast, *SiHAT17* was highly expressed in the shoot, and its expression pattern showed a sharp decline initially, then a gradual increase until returning to its original level at 24 h (Fig. 7).

**Fig. 8:**
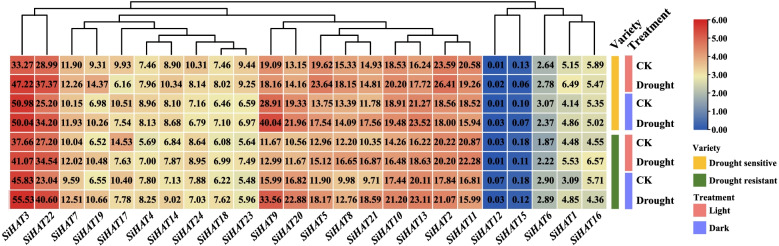
Expression analysis of HATs genes in foxtail millet under drought stress. Control and Drought represented Control and Drought treatment groups, respectively. R (AN04) and S (Yugu1) represent drought-resistant and drought-sensitive varieties, respectively. Light and Dark represent different sampling times and illumination: (light) illumination, (dark) no illumination

Previous studies have reported that the expression of *HAT* genes was induced by drought [37]. Therefore, we analyzed the expression of the 24 *SiHAT*s in our RNA-seq data. The results showed that except for *SiHAT15*, *SiHAT3*, *SiHAT21*, and *SiHAT18*, the other *SiHAT* genes responded to drought stress with different expression patterns. Nine genes (*SiHAT1*, *SiHAT5*, *SiHAT7*, *SiHAT8*, *SiHAT9*, *SiHAT10*, *SiHAT13*, *SiHAT19*, and *SiHAT22*) were upregulated, while *SiHAT17* and *SiHAT15* were downregulated under drought conditions. *SiHAT9* was highly expressed in drought-sensitive foxtail millet varieties. In response to circadian and drought treatments, *SiHAT3*, *SiHAT9*, *SiHAT13*, and *SiHAT20* showed higher expression levels under dark conditions; meanwhile, the remaining five genes (*SiHAT2, SiHAT8, SiHAT11, SiHAT23*, and *SiHAT24*) showed lower expression (Fig. 8).

**Fig. 9:**
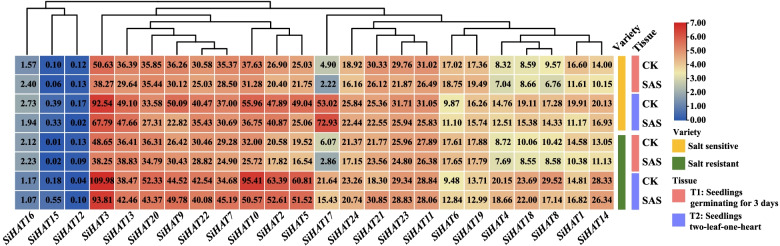
Expression analysis of HATs genes in foxtail millet under salt-alkali stress. CK and SAS represent control group and salt-alkali treatment group, respectively. R (B103) and S (B355) represent salt-resistant and salt-sensitive varieties, respectively. T1 and T2 represent different sampling tissues: T1 is Seedlings germinating for 3 days and T2 is one-tip-two-leaf Seedlings

Under salt and alkali stress, *SiHAT6* was upregulated at low levels, while the other genes were significantly downregulated. The transcript levels of most genes were lower at the germination stage than at the two-leaf one-heart stage. Only *SiHAT6* and *SiHAT19* showed high expression in the germination stage. Additionally, sensitive and resistant varieties showed differences in gene expression. At the T2 stage, the expression of 9 genes (*SiHAT2*, *SiHAT3*, *SiHAT4*, *SiHAT5*, *SiHAT8*, *SiHAT10*, *SiHAT14*, *SiHAT18*, and *SiHAT20*) was higher in the salt-resistant variety than in the sensitive variety, while the expression of three genes (*SiHAT13*, *SiHAT17*, and *SiHAT19*) showed the opposite trend (Fig. 9). *SiHAT12* and *SiHAT15* were the only two genes not or minimally expressed under all conditions (Figs. 6, 7, 8 and 9).

### SiHATs involved in *Sclerospora graminicola* infection

**Fig. 10:**
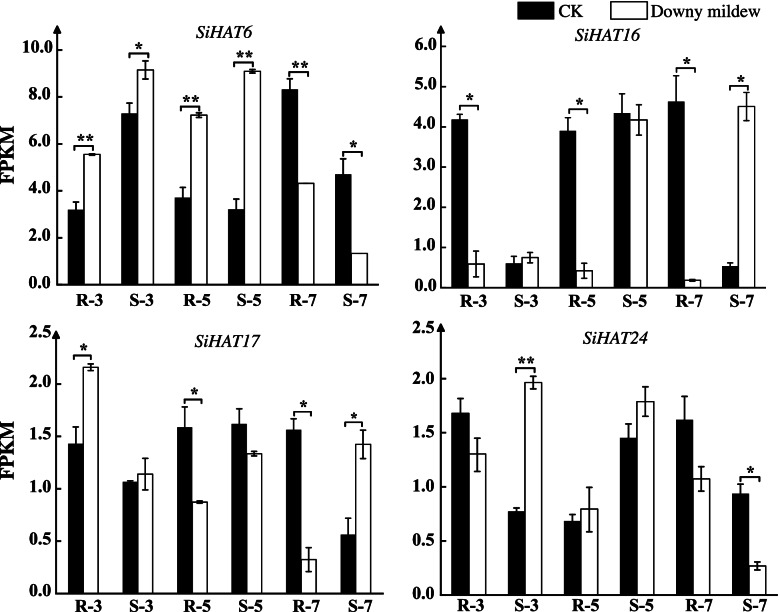
Expression analysis histone acetylation genes (*HAT*s) in *Setaria italica* under *Sclerospora graminicola* infection stress. CK and T represent the control and infection treatment groups, respectively. R and S represent resistant and sensitive varieties, respectively. Numbers 3, 5, and 7 represent leaf stages at different sampling times

The oomycete *S. graminicola* (Sacc.) causes 20–30% of downey mildew cases in foxtail millet cultivated in China and results in the deterioration of yield and quality [37]. It is also prevalent in India, Japan, and Russia [37, 38]. We investigated the expression of *SiHAT*s in our transcriptome sequencing data obtained after *S. graminicola* infection. Four *SiHAT* genes were detected in response to the infection, and their expression patterns were different. During the three-leaf-one-heart stage, *SiHAT16* and *SiHAT24* expressions were downregulated in the pathogen-resistant variety but upregulated in the sensitive variety, while *SiHAT6* and *SiHAT17* expressions were upregulated in both the resistant and sensitive varieties. At the five-leaf-one-heart stage, *SiHAT6* and *SiHAT24* expressions were upregulated after infection, and there was no difference between the sensitive and resistant varieties. At the seven-leaf-one-heart stage, the expression of all four genes were downregulated in the pathogen-resistant variety; however, *SiHAT16* and *SiHAT17* expressions were upregulated in the sensitive variety. The other genes were not or minimally expressed (Fig. 10).

## Discussion

### Characterization of the expanded HAT gene family in foxtail millet

Multiple members of a specific gene family in a particular organism are the natural products generated of a long evolutionary history of that organism [39]. Key chromosome reshuffling events have occurred between foxtail millet, rice, and sorghum [31]. In this study, we identified and characterized 24 foxtail millet *HAT* genes using genome-wide analysis. We found that the foxtail millet *HAT* family is larger than that of *Arabidopsis* [14] and rice with 12 and 19 members, respectively [14, 27], but is smaller than that of wheat with 31 members [40]. The phylogenetic analysis shows that three sister pairs, one triplet, and one quadruplet were identified within the *SiHAT* family. However, none of these pairs were genetically linked to each other, as compared to their corresponding chromosomal locations. Conversely, all closely linked *SiHAT* loci, such as *SiHAT9* and *SiHAT10* on chromosome 2, and *SiHAT17* and *SiHAT18* on chromosome 5, were not paired together into the same sister groups. Moreover, there were no sister pairs mapped on the same duplicated chromosomal blocks (Additional file 1), as described previously. Domains and motifs have been shown to be involved in various activities, including protein interaction, transcriptional activity, and DNA binding; in this study, we found that homologous genes, such as sister pairs and quadruplets, contained conserved motifs. We also found that most *SiHAT* genes shared a similar exon/intron structure within the same phylogenetic group, although some differences were also observed (Fig. 3b). Gene structure analysis can provide important information about gene function and evolution. It has been revealed that intron gain or loss in plants is the result of selection pressures during evolution, and genes tend to evolve into diverse exon-intron structures and perform distinct functions [41]. Therefore, our results suggest that gene differentiation might have occurred in the *SiHAT* family to accomplish different biological functions under selection pressure during foxtail millet genome formation and evolution. Interestingly, clade II is composed of eleven foxtail millet *HATs*, all belonging to the GNAT subfamily, indicating expanding HAT gene events, particularly in the GNAT subgroup of foxtail millet. Genes in this group showed tissue-specific expression, indicating that it may be involved in important processes of growth and development in foxtail millet.

### Expression divergence between duplicated *SiHAT* genes

The presence of duplicated *SiHAT*s raises questions about their functional redundancy. According to evolutionary models, duplicated genes may undergo different selection processes: nonfunctionalization, where one copy loses function; hypofunctionalization, where one copy decreases in expression or function; neofunctionalization, where one copy gains a novel function; or subfunctionalization, where the two copies partition or specialize into distinct functions [42–44]. These evolutionary fates may be indicated by the divergence in expression patterns or protein structure. All *SiHAT*s in this study appeared to be functional because they contained a credible and complete open reading frame, and their corresponding cDNA/ESTs are available in the NCBI database. Evidence for the divergence between the duplicate genes can be inferred from the expression pattern for the HAT quadruplet gene set. *SiHAT2* was highly expressed in leaves and stems; however, the transcript levels of *SiHAT4*, *SiHAT15*, and *SiHAT18* were very low. In addition, possible subfunctionalization trends were clear in the expression pattern shifts of gene pairs. For example, the mRNA abundance of *SiHAT3* peaked in the root at the filling stage; however, *SiHAT20* was highly expressed in the neck panicle internode. Under nitrogen deficiency, phosphorus deficiency, and salt and alkali stress, the expression patterns of *SiHAT3* and *SiHAT20* also differed possibly due to the large differences between the *cis* elements in their promoter.

### Regulation of *SiHAT* gene expression

Accumulating evidence has shown that HAT family genes play pivotal roles in regulating plant growth and development. Arabidopsis *AtHAG1/GCN5* is one of the most widely studied and functionally characterized acetyltransferases and regulates cell differentiation, leaf and floral meristem patterning, and plant defense pathways [45]. In foxtail millet, two *SiHAT*s, *SiHAT17* and *SiHAT23*, were grouped into the same cluster as *AtHAG1/GCN5*. Of these, *SiHAT17* had a similar expression pattern to *AtHAG1*, which is involved in low phosphorus stress, drought, and salt and alkali stress responses. Many identified *HAT*s are involved in seed development; in this study, we found that *SiHAT23* was highly expressed in germinated seeds and contained the endosperm expressive element (GCN4_motif) in its promoter. This indicates the potential role of HAT in seed development and dormancy. A review by Nguyen et al. [20] demonstrated that HAT can collaborate with ARFs to trigger auxin response and regulate root growth and development. Additionally, some *SiHAT*s (*SiHAT3*, *SiHAT9*, *SiHAT13*, and *SiHAT22*) are highly expressed in roots and may play important roles in root development.

### Histone acetylation and abiotic stress responses

The relative expression levels of *HAT* genes change significantly under various biotic and abiotic stresses [16]. *GCN5*, which is involved in salt stress response, was first characterized in maize roots, where its expression increased under salt stress conditions [46]. Similar patterns of expression were detected in *Arabidopsis* under salt stress conditions [47], and the expression of *GCN5* homologous genes *SiHAT17* and *SiHAT23* were also upregulated under salt stress in our experiment. *OsHAC701* has been reported to respond to salt treatment in rice [27], and its homologs, *SiHAT*4 *and SiHAT18*, were also differentially expressed under salt and alkali stress in this study, especially at the seeding of one-heart-two-leaf. This suggests that it may play an important role in salt stress response in foxtail millet. In rice, four histone acetyltransferases, *OsHAC703*, *OsHAG703*, *OsHAF701*, and *OsHAM701*, were involved in drought responses by hyperacetylating lysine residues evident by their upregulation [48]. In our study, their closest homologs also responded to the drought stress, suggesting that the two likely perform the same functions against drought stress in foxtail millet.

### Histone acetylation in nitrogen and phosphorous stress responses

Nitrogen and phosphorus are two major mineral nutrients required for plant growth and development. Histone modifications have been reported in responses to nitrogen levels. *AtHNI9*, which encodes a component of the RNA polymerase II complex (IWS1), plays an important role in modulating the low nitrogen response in *Arabidopsis* [49]. *ZmCHB101* encodes a core subunit of the SWI/SNF-type ATP-dependent chromatin remodeling complex, which can bind to the promoter of the nitrogen transporter *ZmNRT2.1/2.2* through nitrate-responsive *cis*-elements and regulate nitrogen transport. Additionally, its RNAi lines display accelerated root growth and increased biomass under low nitrogen conditions compared to that in wild-type plants [50]. This suggests that histone modification plays an important role in regulating plant growth and development under low nitrogen conditions. In foxtail millet, we found that some *HAT* genes were responsive to low nitrogen stress, and the expression patterns differed between the different groups, indicating that the mechanism of histone regulation is complex, and further investigation is needed to clarify the function of *SiHATs* under low nitrate responses.

The histone acetyltransferase *AtGCN5* is required for the activation of several genes under low Pi conditions, including *At*4 and *AtWRKY6*. Mutation of *AtGCN5* reduces Pi concentration in *Arabidopsis* plant and impairs Pi accumulation between roots and shoots. This indicates that GCN5-mediated histone acetylation regulates the phosphate starvation response through the At4-miR399-PHO2 pathway [51]. We found that the GCN5 homolog gene *SiHAT17* responded to low phosphorus stress and was highly expressed in roots under low phosphorus conditions. However, the expression pattern was reversed in the shoot, suggesting a difference in histone modification between shoot and root under low phosphate conditions in foxtail millet. These findings prove that the mechanisms regulating the responses of shoots and roots to low phosphate stress are different, and further investigation should be conducted on the role of *SiHAT17* in the regulation of low phosphorus stress.

### Histone acetylation and biotic stress responses

Histone acetylation dynamics by HATs and HDACs is a key regulatory epigenetic mechanism that ultimately regulates plant development, hormone homeostasis, stress response, and defense responses in plants [3, 52–55]. In this study, we identified 24 HATs in foxtail millet and found more abiotic and biotic responsive elements in their promoters; and most *SiHAT* genes responded to nitrogen deficiency, phosphorus deficiency, drought, salt alkali stress, and *S. graminicola* infection. All these favorable conditions indicate that these proteins may be involved in plant responses to abiotic and biotic stress; however, the mechanisms behind this stress response, and how HATs interact with other histone modifiers, remain unclear. Future studies should include transgenic research and protein interaction studies on key genes related to abiotic and biotic stress, and the use of new CRISPR-Cas9 technology.

## Conclusions

Recent studies show that histone acetylation and deacetylation play essential roles in the regulation of plant growth and development, as well as in responses to stress. Analyses of various mutations of *HAT* and *HDAC* genes in *Arabidopsis* have revealed the function of histone acetylation/deacetylation in plant development. Here, we screened 24 *HAT* genes that may be involved in abiotic and biotic stress responses in foxtail millet. We identified their chromosomal locations, protein structures, gene duplications, promoters, and conserved motifs. Phylogenetic and synteny comparisons between *AtHAT*s, *OsHAT*s, and *SiHAT*s were performed, and the potential roles of SiHATs in the development and growth of foxtail millet were investigated based on our previously published RNA-seq data. Candidate *SiHAT*s involved in responses to low nitrate, low phosphate, drought, salt response, and *S. graminicola* infection were examined and assessed through the analysis of promoter elements. Our results lay a foundation for research regarding histone modification mediated stress response regulation in foxtail millet and similar crops. Further studies, including biological experiments, will be required to confirm the functions of the candidate genes, identify the interacting *HATs* and *HDACs*, and clarify the molecular mechanisms by which histone acetylation/deacetylation affects various biological processes. This study serves as a reference to improve the stress resistance of plants.

## Methods

### Sequence retrieval and identification of *HAT* genes

We retrieved data containing sequence IDs, protein sequences, genomic sequences, and conserved domain database (CDD) of foxtail millet and rice from Phytozome (https://phytozome.jgi.doe.gov/pz/portal.html V12.1). The *AtHAT* gene sequence from *Arabidopsis thaliana* was retrieved from Uniprot (https://www.uniprot.org), and the Hidden Markov Model was used to identify foxtail millet genes in a protein database with the BLASTP program (*p* value = 0.001). The obtained proteins were run through the Pfam database and SMART (http://pfam.xfam.org/) to eliminate the sequences not containing complete HAT domains. Then, the conserved domains were checked by the conserved domain database (CDD) program (https://www.ncbi.nlm.nih.gov/Structure/cdd/wrpsb.cgi) in order to confirm the presence of the complete HAT domain. Then, all candidate sequences were verified using BLASTn (https://blast.ncbi.nlm.nih.gov/Blast.cgi) and HMMER (http://www.hmmer.org). Finally, the cis-acting elements were predicted using PlantCARE (http://bioinformatics.psb.ugent.be/webtools/plantcare/html/).

### Phylogenetic analysis of *HAT*s

The exon/intron structures of the *SiHAT*s were deduced from alignments of cDNA and BAC genomic sequences using the gene structure displayer (http://gsds.cbi.pku.edu.cn/). Multiple-sequence alignments of SiHAT proteins were carried out using the Clustal W (version 2.0) program [56]. The protein sequences of histone acetyltransferase proteins in *Arabidopsis* and rice were obtained from the TIGR database. Then, phylogenetic analysis was performed with MEGA7.0 [57] using the neighbor-joining method (1000 bootstrap replications), and the results were visualized in Itol [58].

Swiss-Prot, physicochemical properties, subcellular localization, pI, and Mw of the putative SiHATs were calculated using the ExPASy online tool (https://www.genscript.com/psort.htm). Secondary protein structures were predicted using SOPMA software (https://prabi.ibcp.fr/htm/site/web/home).

Chromosome information was obtained from Phytozome (http://www.phytozome.net), and a chromosomal location map of the genes was generated by TBtools (https://github.com/CJ-Chen/TBtools). A distinctive name was given to each SiHAT according to its initial position on the chromosomes. Conserved motif analysis of foxtail millet HAT protein sequences was conducted using the MEME suite 4.11.1 software (http://meme.nbcr.net/meme/) [59], with the motif width set to 6–300 and the maximum number of motifs set to 20. The results were visualized using TBtools.

### Analysis of duplication and synteny of HAT family genes in *Arabidopsis*, foxtail millet, and rice

To confirm the gene duplication events of HATs, we investigated the ancient duplication events between *A. thaliana*, *O. sativa*, and *S. italica*. The non-synonymous substitution rate (Ka), synonymous substitution rate (Ks), and Ka/Ks ratio for each pair of duplicated genes among *A. thaliana, O. sativa*, and *S. italica* were computed between pairs of homologous genes using the Ka/Ks calculator in TBtools with default settings. Multiple collinear scanning was used to simultaneously detect the homologous genetic relationships between different species.

### Promoter analysis of SiHAT genes

The *cis-*acting elements of promoters are essential for determining tissue-specific expression and are involved in the regulation of gene expression under abiotic stress. A region approximately 2000 bp upstream of the start codon (ATG) was investigated from the reference genome sequence of foxtail millet. The online software PlantCARE (http://bioinformatics.psb.ugent.be/webtools/plantcare/html) was used to search for *cis*-acting regulatory elements in the promoters of *SiHATs*.

### Analysis of gene expression profiles

Data on the expression of *SiHAT* genes in 23 tissue types of the foxtail millet cultivar JG21 were obtained from a published foxtail millet database (http://foxtail-millet.biocloud.net/home) [32]. These tissues included leaves at the seeding and different filling stages, seeds at various maturation stages, roots at the filling stage, panicles, and neck panicles, as detailed in Additional file 10. Detailed information can be found in Additional file 9. All RNA sequencing library preparations were conducted as described in previous study [32]. The time and spatial expression data for *SiHAT*s were collected from our Electronic Fluorescent Pictograph (xEFP) program (http://sky.sxau.edu.cn/MDSi.html) and the aforementioned database [32]. Heatmap Illustrator HemiI v.1.0, was used to plot the heat map of gene expression [60].

### Tissue preparation

Seeds from the foxtail millet cultivar Jingu21 were germinated on paper rolls for three days, then incubated at 25 ℃ (day) or 22 ℃ (night) on a 16: 8 h light: dark schedule for three weeks. Seedlings at the five-leaf stage were transferred to Hoagland solution with 1 mM KH_2_PO_4_ (Control, CK) or 5 µM KH_2_PO_4_ (low phosphorus treatment). The shoot and root tissues were harvested at 0.5, 2, 6, 12, 24, and 72 h after treatment.

For the nitrate experiment, the plants were prepared as before. The nitrate concentration was 2.0 mM in the control treatment, (normal nitrate; NN), and 0.2 mM in low nitrate (LN). After 10 min, 30 min, and 2, 8, 24, and 72 h, the shoot and root tissue were harvested. Special care was taken to characterize the materials used, and three replicates were harvested for RNA extraction.

The seedlings were grown in distilled water for 14 d, then subjected to remaining stress treatments. For the drought treatment, two different varieties of foxtail millet were used: AN04 (drought resistant) and Yugu 1 (drought sensitive). Conditions included different circadian stages as morning (8:00–9:00), noon (12:00–13:00), evening (18:00–19:00). Leaf tissues from plants under the control and drought conditions were collected in the morning, noon, and evening after three days of treatment.

For the salt and saline treatments, salt-sensitive (B355) and salt-tolerant (B103) seeds were germinated in Hoagland nutrient solution supplemented with 200 mM NaCl and a 30 mM mixture of Na_2_CO_3_ and NaHCO_3_ to represent salt and saline-alkaline stress; they were germinated in distilled water as the control treatment. Plants were harvested after 3 d (at the germination stage) and two weeks (two-leaf one-heart stage).

For the *S. graminicola* infection experiments, Jingu21 (pathogen-sensitive) and Jingu42 (pathogen-resistant) cultivars were germinated as described for the phosphate experiments. They were then infected at three growth periods: the 3-leaf, 5-leaf, and 7-leaf stages. The uninfected plant was set as the control, and three replicates were harvested for each genotype between 8:00 and 9:00 am.

## Supplementary Information

**Additional file 1:** **Table S1.** Basic physicochemical properties, Secondary structure prediction and subcellular localization of HATs in *Setaria italica*.

**Additional file 2: Table S2.** Information of histone acetylation gene (*HAT*) structure and conserved domain in foxtail millet.

**Additional file 3: Fig. S1. **Phylogenetic trees and domain composition of GNAT subfamily. Phylogenetic tree and domain composition of GNAT subfamily predicted proteins from Arabidopsis thaliana (At),Oryza sativa (Os) and Setaria italica (Si). Conservative domains include Bromo_plant1/Bromodomain superfamily/Bromodomain/Bromo_gcn5_like, BET/BET superfamily, ZipA superfamily, PHA03247 superfamily, COG5076 superfamily, Hat1_N, NAT_SF and ELP3 superfamily.

**Additional file 4: Fig. S2. **Phylogenetic trees and domain composition of CBP subfamily. Phylogenetic tree and domain composition of CBP subfamily predicted proteins from Arabidopsis thaliana (At),Oryza sativa (Os) and Setaria italica (Si). Conservative domains include HAT_KAT11 superfamily, PHD_HAC_like/PHD_SF superfamily, zf-TAZ/ZnF_TAZ/ZnF_UBR1, ZZ superfamily/ZZ_CBP/ZZ_dah/ZZ, Med15 superfamily, BTB_POZ superfamily, BACK and E3_UbLigase_R4.

**Additional file 5: Fig. S3. **Phylogenetic trees and domain composition of MYST subfamily. Phylogenetic tree and domain composition of MYST subfamily predicted proteins from Arabidopsis thaliana (At), Oryza sativa (Os) and Setaria italica (Si). All members of MYST subfamily have a conserved domain PLN00104.

**Additional file 6: Fig. S4. **Phylogenetic trees and domain composition of TAF subfamily. Phylogenetic tree and domain composition of TAF subfamily predicted proteins from Arabidopsis thaliana (At), Oryza sativa (Os) and Setaria italica (Si). Conservative domains include DUF3591/DUF3591 superfamily, Bromodomain superfamily/Bromodomain/Bromo_AAA, Ubiquitin_like_fold superfamily, TBP-binding superfamily, zf-CCHC_6 superfamily, P-loop_NTPase superfamily and SpoVK.

**Additional file 7: Fig. S5. **Phylogenetic trees and three dimensional structures of GNAT proteins in Setaria italica. Bootstrap values higher than 50% are shown. In the same subfamily, the three dimensional structure is similar. The higher the bootstrap values, the closer the kinship and the more similar the three dimensional structure.

**Additional file 8: Table S3. **One-to-one orthologous relationships between *Setaria italica (Si) *and *Oryza sativa(Os)*.

**Additional file 9: Table S4. **Prediction of cis-acting elements in promoters ofhistone acetylation genes (*HAT*s) infoxtail millet.

**Additional file 10: Table S5. **Details of 23tissues sampled in the spatial and temporal expression experiment.

**Additional file 11.  **HATs Protein sequence of  Arabidopsis thaliana, Oryza sativa, and Setaria italica.

## Data Availability

All datasets supporting the results of this study are included within the article and its supplementary information.
