# Supplementary material for: Genome-wide investigation of histone acetyltransferase gene family and its responses to biotic and abiotic stress in foxtail millet (Setaria italica [L.] P. Beauv)
Source: BMC Plant Biol. 2022 Jun 14;22:292. doi: 10.1186/s12870-022-03676-9 (PMC9199193; doi:10.1186/s12870-022-03676-9)
Supplement: Supplementary file 2 — Additional file 2: Table S2. Information of histone acetylation gene (HAT) structure and conserved domain in foxtail millet. [file 12870_2022_3676_MOESM2_ESM.docx]

**Table S2.** **Information of histone acetylation gene (*HAT*) structure and conserved domain in** **foxtail millet.**

| **Name** | **From** | **To** | **Accession** | **Domain** |
| --- | --- | --- | --- | --- |
| SiHAT1 | 338 | 436 | cd05506 | Bromo_plant1 |
|  | 508 | 568 | pfam17035 | BET |
|  | 70 | 274 | cl33720 | PHA03247 superfamily |
| SiHAT2 | 1149 | 1372 | cl02120 | HAT_KAT11 superfamily |
|  | 1018 | 1090 | cd15614 | PHD_HAC_like |
|  | 670 | 739 | pfam02135 | zf-TAZ |
|  | 1608 | 1686 | smart00551 | ZnF_TAZ |
|  | 1549 | 1593 | cl00295 | ZZ superfamily |
|  | 89 | 531 | cl26621 | Med15 superfamily |
|  | 1425 | 1459 | cl00295 | ZZ superfamily |
| SiHAT3 | 167 | 265 | cd05506 | Bromo_plant1 |
|  | 315 | 380 | pfam17035 | BET |
| SiHAT4 | 745 | 966 | cl02120 | HAT_KAT11 superfamily |
|  | 626 | 689 | cl22851 | PHD_SF superfamily |
|  | 1184 | 1254 | pfam02135 | zf-TAZ |
|  | 1020 | 1074 | cl00295 | ZZ superfamily |
| SiHAT5 | 4144 | 5042 | pfam13764 | E3_UbLigase_R4 |
|  | 1535 | 1603 | smart00396 | ZnF_UBR1 |
|  | 2574 | 2625 | cd02249 | ZZ |
| SiHAT6 | 30 | 175 | pfam10394 | Hat1_N |
|  | 217 | 275 | cd04301 | NAT_SF |
| SiHAT7 | 365 | 534 | cl38936 | P-loop_NTPase superfamily |
|  | 880 | 990 | cd05528 | Bromo_AAA |
|  | 383 | 791 | COG0464 | SpoVK |
| SiHAT8 | 122 | 217 | cd04369 | Bromodomain |
| SiHAT9 | 13 | 107 | cd05506 | Bromo_plant1 |
| SiHAT10 | 208 | 304 | cd04369 | Bromodomain |
| SiHAT11 | 35 | 441 | PLN00104 | PLN00104 |
| SiHAT12 | 546 | 1101 | cl37834 | DUF3591 superfamily |
|  | 1601 | 1713 | cl02556 | Bromodomain superfamily |
|  | 25 | 60 | cl07760 | TBP-binding superfamily |
| SiHAT13 | 79 | 180 | cl02556 | Bromodomain superfamily |
|  | 244 | 307 | pfam17035 | BET |
| SiHAT14 | 551 | 1119 | pfam12157 | DUF3591 |
|  | 1649 | 1761 | cl02556 | Bromodomain superfamily |
|  | 634 | 705 | cl28922 | Ubiquitin_like_fold superfamily |
|  | 29 | 69 | cl07760 | TBP-binding superfamily |
|  | 1339 | 1358 | cl21136 | zf-CCHC_6 superfamily |
| SiHAT15 | 990 | 1207 | cl02120 | HAT_KAT11 superfamily |
|  | 857 | 930 | cd15614 | PHD_HAC_like |
|  | 1451 | 1519 | pfam02135 | zf-TAZ |
|  | 527 | 590 | pfam02135 | zf-TAZ |
|  | 1389 | 1432 | cl00295 | ZZ superfamily |
|  | 1265 | 1301 | cl00295 | ZZ superfamily |
| SiHAT16 | 150 | 248 | cl02556 | Bromodomain superfamily |
|  | 259 | 323 | cl25276 | BET superfamily |
| SiHAT17 | 95 | 192 | cl38908 | BTB_POZ superfamily |
|  | 275 | 366 | smart00551 | ZnF_TAZ |
|  | 198 | 257 | cd14733 | BACK |
| SiHAT18 | 746 | 868 | cl02120 | HAT_KAT11 superfamily |
|  | 616 | 679 | cl22851 | PHD_SF superfamily |
|  | 1069 | 1140 | pfam02135 | zf-TAZ |
|  | 903 | 930 | cl00295 | ZZ superfamily |
| SiHAT19 | 263 | 354 | cl02556 | Bromodomain superfamily |
|  | 461 | 604 | cl34554 | ZipA superfamily |
| SiHAT20 | 167 | 264 | cd05506 | Bromo_plant1 |
|  | 373 | 435 | pfam17035 | BET |
| SiHAT21 | 62 | 569 | cl36845 | ELP3 superfamily |
| SiHAT22 | 65 | 159 | cd05506 | Bromo_plant1 |
| SiHAT23 | 406 | 504 | cd05509 | Bromo_gcn5_like |
|  | 148 | 501 | cl34891 | COG5076 superfamily |
| SiHAT24 | 206 | 302 | cd04369 | Bromodomain |
|  |  |  |  |  |
