# Supplementary material for: Genome-wide investigation of histone acetyltransferase gene family and its responses to biotic and abiotic stress in foxtail millet (Setaria italica [L.] P. Beauv)
Source: BMC Plant Biol. 2022 Jun 14;22:292. doi: 10.1186/s12870-022-03676-9 (PMC9199193; doi:10.1186/s12870-022-03676-9)
Supplement: Supplementary file 3 — Additional file 3: Fig. S1. Phylogenetic trees and domain composition of GNAT subfamily. Phylogenetic tree and domain composition of GNAT subfamily predicted proteins from Arabidopsis thaliana (At),Oryza sativa (Os) and Setaria italica (Si). Conservative domains include Bromo_plant1/Bromodomain superfamily/Bromodomain/Bromo_gcn5_like, BET/BET superfamily, ZipA superfamily, PHA03247 superfamily, COG5076 superfamily, Hat1_N, NAT_SF and ELP3 superfamily. [file 12870_2022_3676_MOESM3_ESM.pdf]

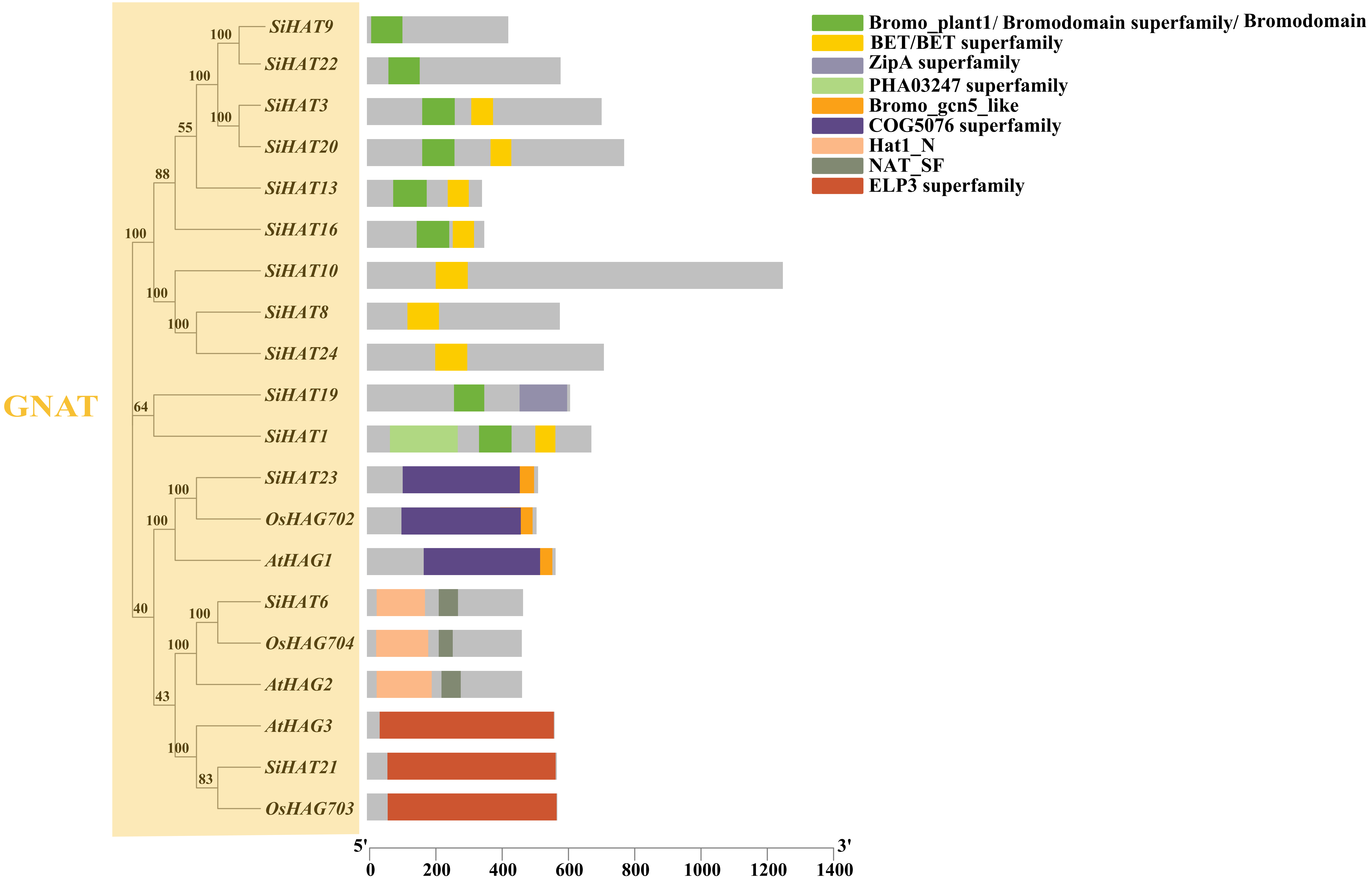

Additional file 3. Phylogenetic trees and domain composition of GNAT subfamily. Phylogenetic tree and domain composition of GNAT subfamily predicted proteins from *Arabidopsis thaliana* (*At*), *Oryza sativa* (*Os*) and *Setaria italica* (*Si*). Conservative domains include Bromo_plant1/Bromodomain superfamily/Bromodomain/Bromo_gcn5_like, BET/BET superfamily, ZipA superfamily, PHA03 247 superfamily, COG5076 superfamily, Hat1_N, NAT_SF and ELP3 superfamily
