# Supplementary material for: Genome-wide investigation of histone acetyltransferase gene family and its responses to biotic and abiotic stress in foxtail millet (Setaria italica [L.] P. Beauv)
Source: BMC Plant Biol. 2022 Jun 14;22:292. doi: 10.1186/s12870-022-03676-9 (PMC9199193; doi:10.1186/s12870-022-03676-9)
Supplement: Supplementary file 4 — Additional file 4: Fig. S2. Phylogenetic trees and domain composition of CBP subfamily. Phylogenetic tree and domain composition of CBP subfamily predicted proteins from Arabidopsis thaliana (At),Oryza sativa (Os) and Setaria italica (Si). Conservative domains include HAT_KAT11 superfamily, PHD_HAC_like/PHD_SF superfamily, zf-TAZ/ZnF_TAZ/ZnF_UBR1, ZZ superfamily/ZZ_CBP/ZZ_dah/ZZ, Med15 superfamily, BTB_POZ superfamily, BACK and E3_UbLigase_R4. [file 12870_2022_3676_MOESM4_ESM.pdf]

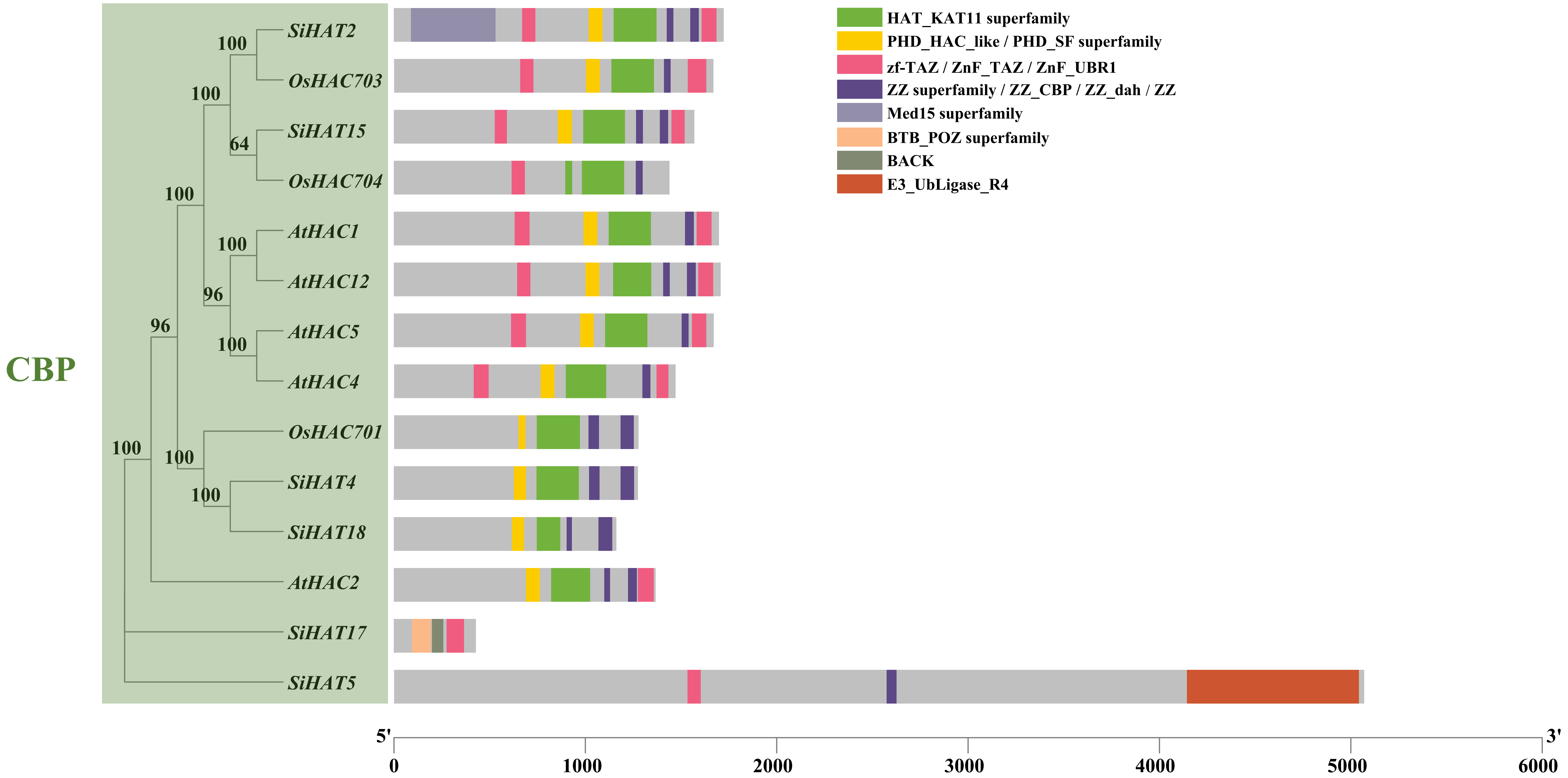

Additional file 4. Phylogenetic trees and domain composition of CBP subfamily. Phylogenetic tree and domain composition of CBP subfamily predicted proteins from *Arabidopsis thaliana* (*At*), *Oryza sativa* (*Os*) and *Setaria italica* (*Si*). Conservative domains include HAT_KAT11 superfamily, PHD_HAC_like/PHD_SF superfamily, zf-TAZ/ZnF_TAZ/ZnF_UBR1, ZZ superfamily/ZZ_CBP/ZZ_dah/ZZ, Med15 superfamily, BTB_POZ superfamily, BACK and E3_UbLigase_R4
