# Supplementary material for: Genome-wide investigation of histone acetyltransferase gene family and its responses to biotic and abiotic stress in foxtail millet (Setaria italica [L.] P. Beauv)
Source: BMC Plant Biol. 2022 Jun 14;22:292. doi: 10.1186/s12870-022-03676-9 (PMC9199193; doi:10.1186/s12870-022-03676-9)
Supplement: Supplementary file 5 — Additional file 5: Fig. S3. Phylogenetic trees and domain composition of MYST subfamily. Phylogenetic tree and domain composition of MYST subfamily predicted proteins from Arabidopsis thaliana (At), Oryza sativa (Os) and Setaria italica (Si). All members of MYST subfamily have a conserved domain PLN00104. [file 12870_2022_3676_MOESM5_ESM.pdf]

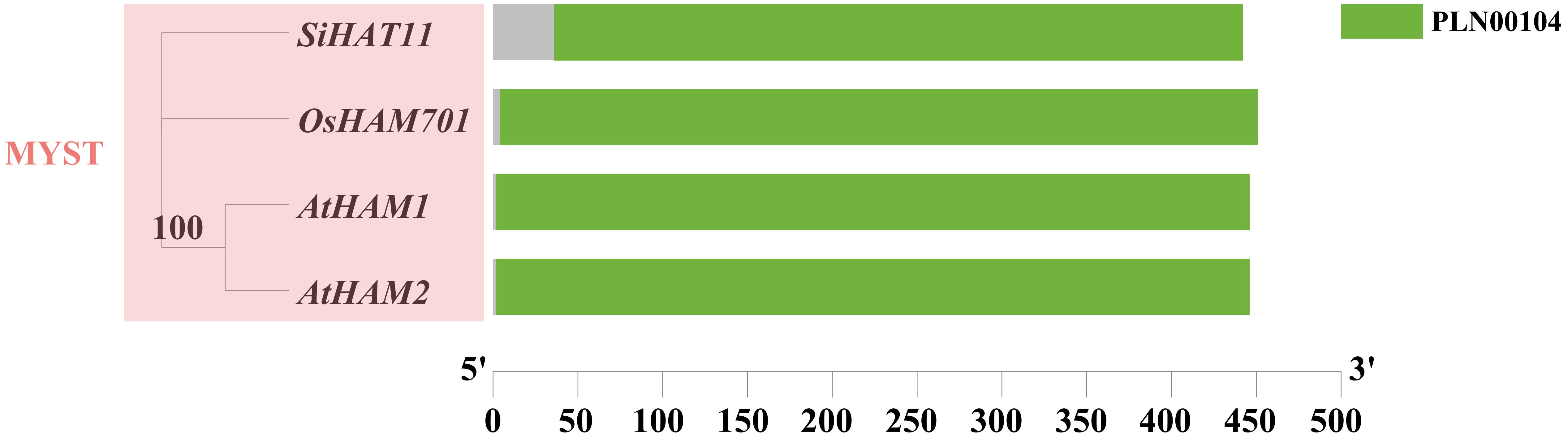

Additional file 5. Phylogenetic trees and domain composition of MYST subfamily. Phylogenetic tree and domain composition of MYST subfamily predicted proteins from *Arabidopsis thaliana* (*At*), *Oryza sativa* (*Os*) and *Setaria italica* (*Si*). All members of MYST subfamily have a conserved domain PLN00104.
