# Supplementary material for: Genome-wide investigation of histone acetyltransferase gene family and its responses to biotic and abiotic stress in foxtail millet (Setaria italica [L.] P. Beauv)
Source: BMC Plant Biol. 2022 Jun 14;22:292. doi: 10.1186/s12870-022-03676-9 (PMC9199193; doi:10.1186/s12870-022-03676-9)
Supplement: Supplementary file 6 — Additional file 6: Fig. S4. Phylogenetic trees and domain composition of TAF subfamily. Phylogenetic tree and domain composition of TAF subfamily predicted proteins from Arabidopsis thaliana (At), Oryza sativa (Os) and Setaria italica (Si). Conservative domains include DUF3591/DUF3591 superfamily, Bromodomain superfamily/Bromodomain/Bromo_AAA, Ubiquitin_like_fold superfamily, TBP-binding superfamily, zf-CCHC_6 superfamily, P-loop_NTPase superfamily and SpoVK. [file 12870_2022_3676_MOESM6_ESM.pdf]

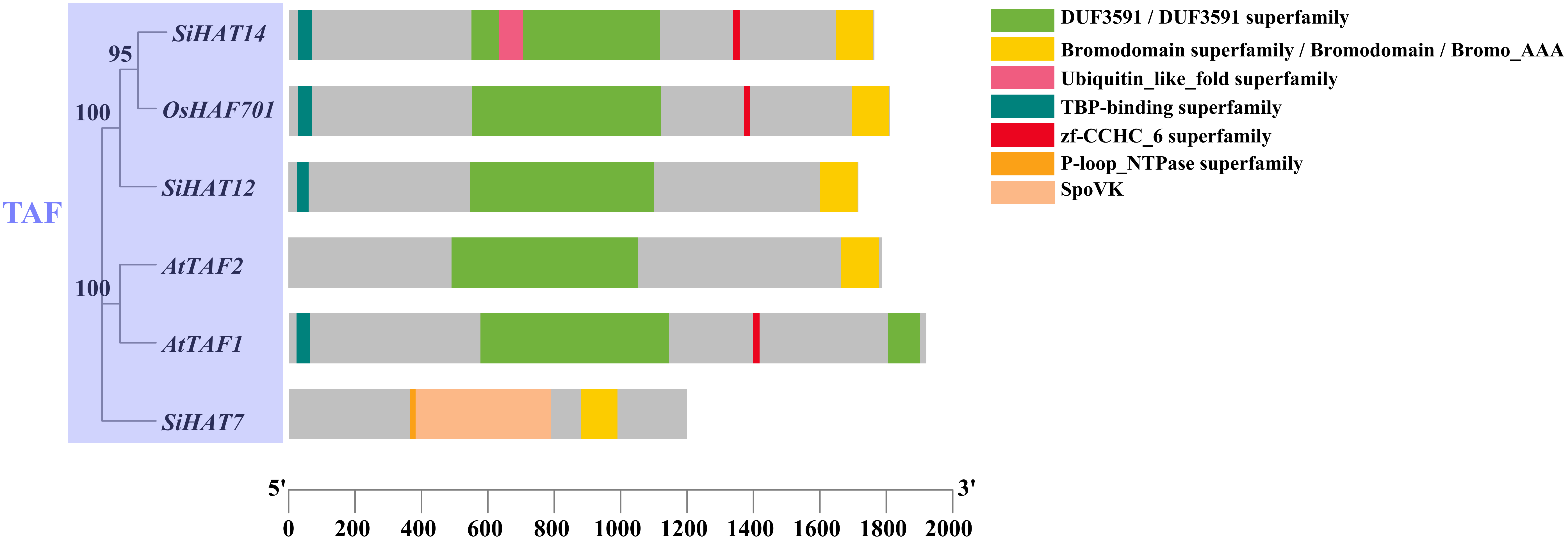

Additional file 6. Phylogenetic trees and domain composition of TAF subfamily. Phylogenetic tree and domain composition of TAF subfamily predicted proteins from *Arabidopsis thaliana* (*At*), *Oryza sativa* (*Os*) and *Setaria italica* (*Si*). Conservative domains include DUF3591/DUF3591 superfamily, Bromodomain superfamily/Bromodomain/Bromo_AAA, Ubiquitin_like_fold superfamily, TBP-binding superfamily, zf-CCHC_6 superfamily, P-loop_NTPase superfamily and SpoVK.
