# Supplementary material for: Genome-wide investigation of histone acetyltransferase gene family and its responses to biotic and abiotic stress in foxtail millet (Setaria italica [L.] P. Beauv)
Source: BMC Plant Biol. 2022 Jun 14;22:292. doi: 10.1186/s12870-022-03676-9 (PMC9199193; doi:10.1186/s12870-022-03676-9)
Supplement: Supplementary file 7 — Additional file 7: Fig. S5. Phylogenetic trees and three dimensional structures of GNAT proteins in Setaria italica. Bootstrap values higher than 50% are shown. In the same subfamily, the three dimensional structure is similar. The higher the bootstrap values, the closer the kinship and the more similar the three dimensional structure. [file 12870_2022_3676_MOESM7_ESM.pdf]

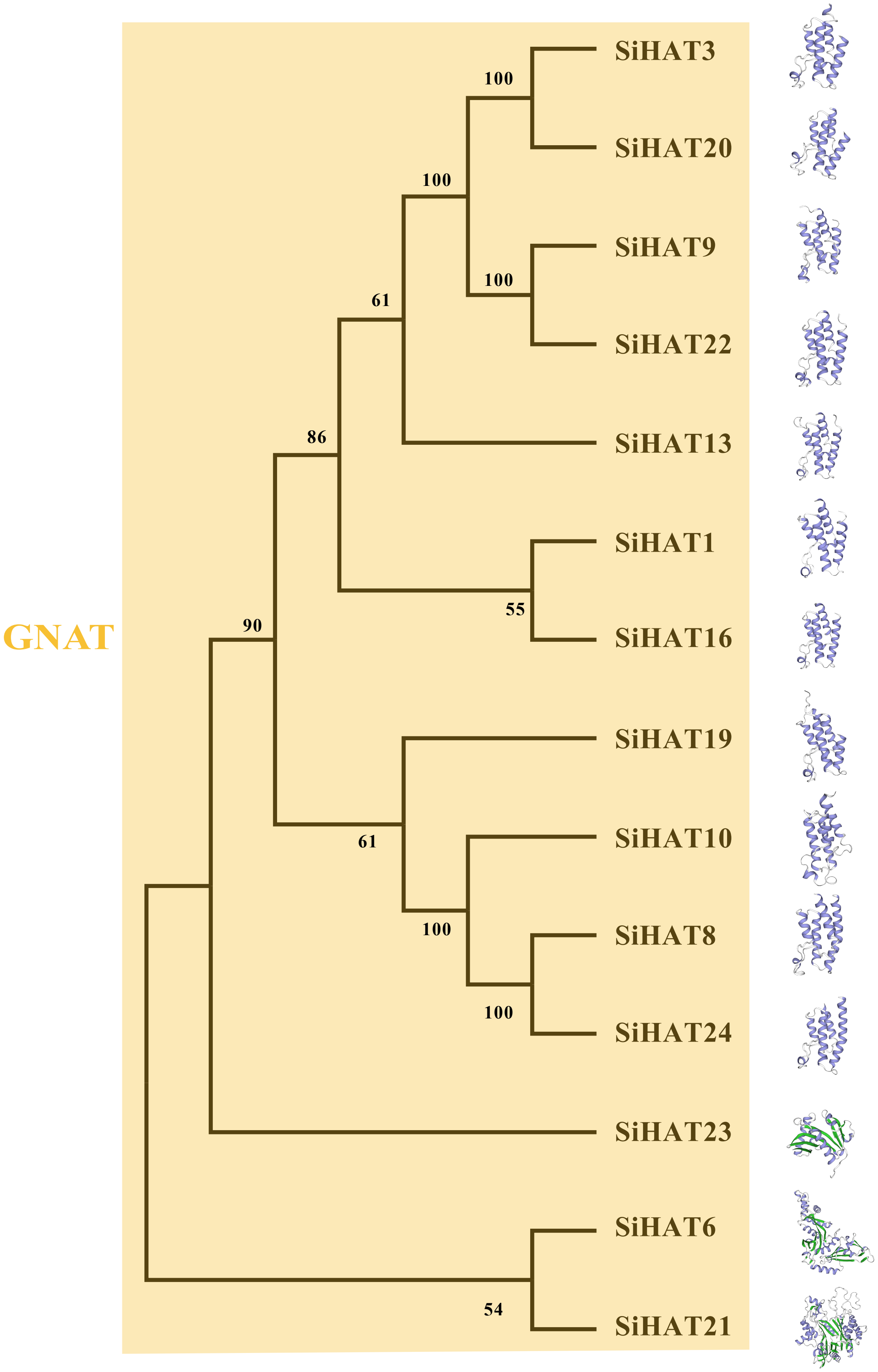

Additional file 7. Phylogenetic trees and three dimensional structures of GNAT proteins in *Setaria italica*. Bootstrap values higher than 50% are shown. In the same subfamily, the three dimensional structure is similar. The higher the bootstrap values, the closer the kinship and the more similar the three dimensional structure.
