# Supplementary material for: Genome-wide investigation of histone acetyltransferase gene family and its responses to biotic and abiotic stress in foxtail millet (Setaria italica [L.] P. Beauv)
Source: BMC Plant Biol. 2022 Jun 14;22:292. doi: 10.1186/s12870-022-03676-9 (PMC9199193; doi:10.1186/s12870-022-03676-9)
Supplement: Supplementary file 8 — Additional file 8: Table S3. One-to-one orthologous relationships between Setaria italica (Si) and Oryza sativa(Os). [file 12870_2022_3676_MOESM8_ESM.docx]

**Table S3. One-to-one orthologous relationships between *Setaria italica (Si)* and *Oryza sativa (Os)*.**

| Gene_1 | Gene_2 | Ka | Ks | Ka/Ks |
| --- | --- | --- | --- | --- |
| SiHAT6 | OsHAG704 | 0.0878581 | 0.60532754 | 0.14514142 |
| SiHAT21 | OsHAG703 | 0.01549108 | 0.47206296 | 0.03281572 |
| SiHAT23 | OsHAG702 | 0.03246596 | 0.60793909 | 0.0534033 |
| SiHAT11 | OsHAM701 | 0.01849323 | 0.51596747 | 0.03584186 |
| SiHAT2 | OsHAC704 | 0.285964 | 1.01574457 | 0.28153141 |
| SiHAT2 | OsHAC703 | 0.09894152 | 0.57044135 | 0.17344731 |
| SiHAT15 | OsHAC703 | 0.29105518 | 1.05454526 | 0.27600065 |
| SiHAT15 | OsHAC704 | 0.31780627 | 0.64520791 | 0.49256412 |
| SiHAT14 | OsHAF701 | 1.1641396 | 2.25213731 | 0.51690437 |
