# Supplementary material for: Genome-wide investigation of histone acetyltransferase gene family and its responses to biotic and abiotic stress in foxtail millet (Setaria italica [L.] P. Beauv)
Source: BMC Plant Biol. 2022 Jun 14;22:292. doi: 10.1186/s12870-022-03676-9 (PMC9199193; doi:10.1186/s12870-022-03676-9)
Supplement: Supplementary file 9 — Additional file 9: Table S4. Prediction of cis-acting elements in promoters ofhistone acetylation genes (HATs) infoxtail millet. [file 12870_2022_3676_MOESM9_ESM.docx]

**Table S4. Prediction of** **cis-acting elements in promoters of histone acetylation genes (*HAT*s) in foxtail millet.**

| Functional class | Elements | Function | Sequence | Genes |
| --- | --- | --- | --- | --- |
| Development | O2-site | zein metabolism regulatory element | GTTGACGTGA | SiHAT2、SiHAT3、SiHAT5、SiHAT6、SiHAT7、SiHAT8、SiHAT17、SiHAT20 |
|  | CAT-box | meristem expressive element | GCCACT | SiHAT1、SiHAT4、SiHAT5、SiHAT6、SiHAT7、SiHAT9、SiHAT12、SiHAT13、SiHAT14、SiHAT15、SiHAT17、SiHAT19、SiHAT20、SiHAT23、 |
|  | circadian | circadian controlled element | CAAAGATATC | SiHAT15 |
|  | GCN4_motif | endosperm expressive element | TGAGTCA | SiHAT4、SiHAT6、SiHAT15、SiHAT23 |
|  | motif I | root specific element | gGTACGTGGCG | SiHAT5 |
|  | RY-element | seed-specific regulatory element | CATGCATG | SiHAT10、SiHAT19、SiHAT22、SiHAT24 |
| hormone | TGACG-motif | the MeJA-responsive element | TGACG | SiHAT1、SiHAT2、SiHAT3、SiHAT4、SiHAT6、SiHAT8、SiHAT9、SiHAT10、SiHAT11、SiHAT12、SiHAT13、SiHAT14、SiHAT15、SiHAT16、SiHAT17、SiHAT18、SiHAT19、SiHAT23、SiHAT24 |
|  | CGTCA-motif |  | CGTCA |  |
|  | ABRE | the abscisic acid responsive element | ACGTG | SiHAT1、SiHAT2、SiHAT3、SiHAT5、SiHAT6、SiHAT7、SiHAT8、SiHAT9、SiHAT10、SiHAT11、SiHAT12、SiHAT13、SiHAT14、SiHAT15、SiHAT17、SiHAT18、SiHAT19、SiHAT20、SiHAT21、SiHAT22、SiHAT24 |
|  |  |  | CGCACGTGTC |  |
|  |  |  | CACGTG |  |
|  |  |  | TACGTGTC |  |
|  |  |  | GACACGTGGC |  |
|  |  |  | AACCCGG |  |
|  |  |  | GCCGCGTGGC |  |
|  |  |  | TACGGTC |  |
|  |  |  | GCAACGTGTC |  |
|  |  |  | CGTACGTGCA |  |
|  | TATC-box | gibberellin-responsive element | TATCCCA | SiHAT1、SiHAT4、SiHAT7、SiHAT8、SiHAT10、SiHAT11、SiHAT12、SiHAT13、SiHAT14、SiHAT15、SiHAT16、SiHAT17、SiHAT18、SiHAT23、SiHAT24 |
|  | GARE-motif |  | TCTGTTG |  |
|  | P-box |  | CCTTTTG |  |
|  | AuxRR-core | Auxin-responsive element | GGTCCAT | SiHAT3、SiHAT4、SiHAT5、SiHAT6、SiHAT8、SiHAT10、SiHAT11、SiHAT12、SiHAT13、SIHAT17、SiHAT19、SiHAT20、SiHAT21 |
|  | TGA-element |  | AACGAC |  |
|  | TGA-box |  | TGACGTAA |  |
|  | TCA-element | salicylic acid responsive element | TCAGAAGAGG | SiHAT1、SiHAT5、SiHAT8、SiHAT12、SiHAT14、SiHAT16、SiHAT17、SiHAT18、SiHAT21、SiHAT22 |
|  |  |  | CCATCTTTTT |  |
| stress | TC-rich repeats | defense and stress responsive element | ATTCTCTAAC | SiHAT2、SiHAT4、SiHAT11、SiHAT12、SiHAT13、SiHAT15、SiHAT16、SiHAT20、SiHAT21、SiHAT22、 |
|  |  |  | GTTTTCTTAC |  |
|  | LTR | low-temperature responsive element | CCGAAA | SiHAT2、SiHAT5、SiHAT7、SiHAT8、SiHAT17、SiHAT20 |
|  | MBS | MYB binding site involved in drought-inducibility | CAACTG | SiHAT1、SiHAT3、SiHAT5、SiHAT6、SiHAT7、SiHAT8、SiHAT14、SiHAT15、SiHAT17、SiHAT18、SiHAT19、SiHAT20、SiHAT21、SiHAT22、SiHAT23、SiHAT24、 |
|  | WUN-motif | wound-responsive element | AAATTTCCT | SiHAT10 |
| others | GATA-motif | light responsive element | AAGGATAAGG | SiHAT1、SiHAT2、SiHAT3、SiHAT4、SiHAT5、SiHAT6、SiHAT7、SiHAT8、SiHAT9、SiHAT10、SiHAT11、SiHAT12、SiHAT13、SiHAT14、SiHAT15、SiHAT16、SiHAT17、SiHAT18、SiHAT19、SiHAT20、SiHAT21、SiHAT22、SiHAT23、SiHAT24 |
|  | AE-box |  | AGAAACAA |  |
|  | Sp1 |  | GGGCGG |  |
|  | Box 4 |  | ATTAAT |  |
|  | G-Box |  | CACGTG |  |
|  | ACE |  | CACGTC |  |
|  | TCCC-motif |  | GACACGTATG |  |
|  | ATCT-motif |  | AGAAACTT |  |
|  | I-box |  | TCTCCCT |  |
|  | TCT-motif |  | CACGTT |  |
|  | GT1-motif |  | CACGAC |  |
|  | Box II |  | TACGTG |  |
|  | 3-AF1 binding site |  | AATCTAATCC |  |
|  | chs-CMA2a |  | gGATAAGGTG |  |
|  | ATC-motif |  | ccttatcct |  |
|  | GTGGC-motif |  | GATAAGGGT |  |
|  | GATT-motif |  | TCTTAC |  |
|  | AT1-motif |  | TAAACGTG |  |
|  | chs-CMA1a |  | CTTCCACGTGGCA |  |
|  | GA-motif |  | tgACACGTGGCA |  |
|  |  |  | GCCACGTGGA |  |
|  |  |  | GGTTAAT |  |
|  |  |  | GGTTAA |  |
|  |  |  | TAACACGTAG |  |
|  |  |  | ACACGTAGA |  |
|  |  |  | TAAGAGAGGAA |  |
|  |  |  | TCACTTGA |  |
|  |  |  | CTAACGTATT |  |
|  |  |  | AGTAATCT |  |
|  |  |  | GATAGGA |  |
|  |  |  | GATAGGG |  |
|  |  |  | GCGACGTACC |  |
|  |  |  | cGATAAGGCG |  |
|  |  |  | CATCGTGTGGC |  |
|  |  |  | AGATAAGG |  |
|  |  |  | CTCCTGATTGGA |  |
|  |  |  | CCACGTGGC |  |
|  |  |  | GCGGTAATT |  |
|  |  |  | GATTCTGTGGC |  |
|  |  |  | AATTATTTTTTATT |  |
|  |  |  | TTACTTAA |  |
|  |  |  | ATAGATAA |  |
|  | GC-motif | anoxic specific inducibility element | CCCCCG | SiHAT1、SiHAT2、SiHAT3、SiHAT4、SiHAT5、SiHAT6、SiHAT7、SiHAT9、SiHAT10、SiHAT11、SiHAT12、SiHAT13、SiHAT14、SiHAT15、SiHAT16、SiHAT19、SiHAT20、SiHAT23 |
|  | MBSI | MYB binding site involved in flavonoid biosynthetic genes regulation | aaaAaaC(G/C) GTTA | SiHAT2 |
|  |  |  | TTTTTACGGTTA |  |
|  | 3-AF3 binding site | part of a conserved DNA module array (CMA3) | CACTATCTAAC | SiHAT2、SiHAT15 |
|  | CCAAT-box | MYBHv1 binding site | CAACGG | SiHAT1、SiHAT4、SiHAT5、SiHAT8、SiHAT11、SiHAT12、SiHAT13、SiHAT14、SiHAT15、SiHAT16、SiHAT20、SiHAT21、SiHAT23 |
|  | ARE | the anaerobic inductive element | AAACCA | SiHAT1、SiHAT3、SiHAT4、SiHAT5、SiHAT6、SiHAT7、SiHAT9、SiHAT10、SiHAT12、SiHAT13、SiHAT14、SiHAT15、SiHAT18、SiHAT20、SiHAT21、SiHAT22、SiHAT24 |
|  | AT-rich sequence | element for maximal elicitor-mediated activation (2copies) | TAAAATACT | SiHAT7、SiHAT18 |
|  | AT-rich element | binding site of AT-rich DNA binding protein (ATBP-1) | ATAGAAATCAA | SiHAT12 |
|  | MRE | MYB binding site involved in light responsiveness | AACCTAA | SiHAT3、SiHAT14、SiHAT18、SiHAT20 |
