# Supplementary material for: Genome-wide investigation of histone acetyltransferase gene family and its responses to biotic and abiotic stress in foxtail millet (Setaria italica [L.] P. Beauv)
Source: BMC Plant Biol. 2022 Jun 14;22:292. doi: 10.1186/s12870-022-03676-9 (PMC9199193; doi:10.1186/s12870-022-03676-9)
Supplement: Supplementary file 10 — Additional file 10: Table S5. Details of 23tissues sampled in the spatial and temporal expression experiment. [file 12870_2022_3676_MOESM10_ESM.docx]

**Table S5. Details of 23 tissues sampled in the spatial and temporal expression experiment**

| Tissue | Sample | Stage | Description |
| --- | --- | --- | --- |
| leaf | Germinated seeds | Seedling stage | germinated seedlings for 3d |
|  | Seedling |  | two-leaf one-heart of seedlings |
|  | Leaf top2-3 | Heading stage | 2 and 3 top leaves two days after heading |
|  | Flag leaf | Filling stage | flag leaf at the filling stage |
|  | Leaf top-fourth |  | fourth leaf at the filling stage |
|  | Leaf veins |  | Leaf veins at the filling stage |
|  | Mesophyll |  | Mesophyll at the filling stage |
| Stem | Neck panicle internodes | Filling stage | neck panicle-internodes at the filling stage |
|  | Flag leaf sheath |  | leaf sheath at the filling stage |
|  | Stem top-second |  | second section of stem at the filling stage |
|  | Leaf sheath top-fourth |  | fourth parietal leaf sheath at the filling stage |
| root | Root | Filling stage | roots at the filling stage |
| Spike | Panicle 1 | Primary panicle branch differentiation stage | the panicle at the initial spike branching and differentiation stage |
|  | Panicle 2 | Third panicle branch differentiation stage | the panicle of the third panicle branching differentiation stage, |
|  | Immature-spikelet S2 | Filling stage | immature spikelet at S2 and S4 stage, |
|  | Immature-spikelet S4 |  |  |
|  | Immature-seed S1 |  | immature seeds of S1-S5 stage |
|  | Immature-seed S2 |  |  |
|  | Immature-seed S3 |  |  |
|  | Immature-seed S4 |  |  |
|  | Immature-seed S5 |  |  |
