# Supplementary material for: Genome-wide investigation of histone acetyltransferase gene family and its responses to biotic and abiotic stress in foxtail millet (Setaria italica [L.] P. Beauv)
Source: BMC Plant Biol. 2022 Jun 14;22:292. doi: 10.1186/s12870-022-03676-9 (PMC9199193; doi:10.1186/s12870-022-03676-9)
Supplement: Supplementary file 11 — Additional file 11. HATs Protein sequence of Arabidopsis thaliana, Oryza sativa, and Setaria italica. [file 12870_2022_3676_MOESM11_ESM.docx]

>SiHAT1

MHGLAPPGGGVANWEEAAAAGPAPASSTAPSAPPRSRWASPIKVYYRKYPRRNPKPPPPP

PPGPAPDLNPLPETLSSIPRPIRCPEDGVAAAAPSSTPPPFPDPAATSSTTPPFPDPAAP

TSSSPAPPLAPAPPGEPSPGSGDIAPGINRDGAAVPNGHGKDWAMVAVQKAEKAKKRRAR

SELRRQLASELDQVRMLSKRLKAAAETLAQQEDSVPMPLAMMLPPSQVVDARYIRSQFSP

TGPVTPIPAQAAFAPFRSLPPQAPLTVSVVHTEPFEVEKRTPKANQLYQNSEFLLAKDKF

PPADTHGRKKPKHHKKKNRSLEPRGADFDAERRLYSHAFKKSSSLLSRLMKHKFAWVFNK

PVDAVGLGLHDYFEIIRHPMDLGTIRGRLSHGQYRNPKEFAEDVRLTFQNAMTYNPKGHD

VHIMAEQLLGIFEAQWPEIEAKVNYLALCPPLPKKFPPPPIDLRLLERSDSVKHHVALES

NSRPISHTPTRPPSMKKPRAKDANKRDMTMDEKRKLSENLQNLPPEKLDAVVQLIKNKNL

TVRQHDDEIEVEIDSMDAETLWELDRFVSNYKKNLSKQKRRAERAMLARQDAELRAQHSV

QQLLAEERNTGEKSPKQNVMVGGQLASSAPNQNDNNGHVASRSNSSSGSSSDSGSSSSDS

DSDSSSSDGSDAANLS*

>SiHAT3

MTPTVLMEFGQQRPIKRGYEEMAFRGVAAAAPRGYSETVGESEGAAGSPVRVDSEVSAAP

KRKCISLNSDGFDVKREIFVPSKMSSSERRYLRKRFRAELDSVRDLLKKPAFAAPAPVSR

APALSSSAAPRAKKLHRGTNVIRGAKGRFLPTKPRPEPSVELSEAAVFKQCEAILKKLMT

QKYSHIFNIPVDVVKLQIPDYFDIIKTPMDLGTVQKKLESGSYTSPSDFAADVRLTFNNA

MTYNPRGHAVHDMAIQLNKMFENRWRTVEKKLASAAIEKHVDVDKADSKRRKTPPVDRSD

VSVEGLRQTEPVKPKMTAAEREAFGNSLAEIADDLPAHIVELLQQCMDSNTDTAGDGEIE

IDIQAVSDDLLFELKKQVDKYLQEREQNQQVKSEPSENEAVNVSGLSHSSTNPCKGGEPI

EEDVDICGNASPIMLDKDAQLRSNKCVSPSSSSSESESSSSDSDSGSDSESESEKVGSPA

KLAKGPKKPDQLVEQEKSDVISPADVNCPADIVGLREEDSESKPAPEGENSKPDTQVSPD

RLLRAAVLRSRYADVIVKARGILSQGGDKQEELEKLQKEEKARLLAEGNAAMEARRAEAE

AEAKRKRDFEREKARQALQEMERTVEINDNLHLKDLEMLGTATAEHIVSSVDETSPEHSQ

DCMPGFLPGSVNPLEQLGLFMKADEEEDDEEPSSVPSVKEAEEGEIN*

>SiHAT6

MALKQKGTDAAAAADPKKRRRVGFSGIDAGVEANECMKVFLARNSDEVGSEDCTSIQPFD

LNHFFGEDGKIYGYKNLKINVWISAISFHGYADISFDETSDGGKGITDLNTVLQSIFGES

LVEKEEFMQTFSKECEYIRDVVTNGSAIKHNGTNESDPAVEIVRVELQGVAAFLYSRLVP

LVLLLVEGSTPIDIGEHGWEMLLVVKRTTQESVSKFQLLGFAAVHNFYHYPESNRLRISQ

ILVLPPHQGEGHGLRLLEAINSIAQSENIYDVTIEDPSDYLQYVRSSIDCLRLLTLDPIK

PALSAMVSSLKETNLSKRTCSLKMVPPADLTETVRQKLKINKKQFLRCWEILIYLSLDSE

DRKSMDNFRACIYDRTKGEILGGATGTNGKRLVQMSSSVNEEVSFAVYWTQEGGDADDQT

VEQQPEDLKTQEQQLNELVDNQMEEIVGVAKNVSSRVLPVGPQAYCEFLR*

>SiHAT9

MAESMNVSKTRLCGKILKKLMDHKGGWLFHKPVDPVLYGIPDYFDVICNPMDLGTVKKKL

TNKQYVTASEFAADVRLTFANAMKYNPPGNDVHAVAEQLNRIFDSEWRSVIRKWNGRNPV

QEQKPMKATKPEAAMKSKSLIPRGLVTCSNSLAKEPSINAISSKVKIKFFVRGCENTSLK

AGIQEHSIDNSLDCTKGNDKISRIQSNESCALSNGNESPSCNSTSPLASCEQGEESYLHL

EPLSPSKALRIAMLKSRFAGTIVKAQQNALLDHGKEVDLAKLQLEKERLEKSQLEEKARI

EAQVKAAEAAAQLKLEEEMRMKREKERKAARLALHMMKKTVDIDNSDFLKDLENLCQKWQ

LNPPSKLIVDFVHGIELPQGLGSPLEALGLFIKKDLEEEVEQELEDSLSTSLNADVEEGE

ISCCQ*

>SiHAT13

MTPANGAAAPEAVAEAGLAPEVESEADAFQRQVDDLVSKTDVLERRVNEVVDFYDGKKHG

SGGRKGGRHGPHPRGMPDLMRQFGVVLREITSDKKAWPFREPVDVVGMNLHDYYKIITKP

MDFSTIQNKMEGKDVTTYKNVREIYADVRLIFANAMKYNDEENVVHLMAKSLLEKFEEKW

LQFLPKVESEEKRQKDEESKGVVSTSTSREAAIAKLAKDTDDELNQINKQLEELRKMVVN

RCRKMTTDEKRKLGAGLCHLSPDDLNKALEIVAQDNPSFQTKAEEVDLDMDAQSETTLWR

LKFFVREALERQANVASGKMDENAKRKREICNALAKTASKRIKKQP*

>SiHAT16

MPRSTRPPPGFFDESSHPRRRTDAPSSGGGAGGAPVGLESGAGRGILPRPSMKPQNPPVS

RPKRPPPGFFDESRHLRRRTDAPSSGGGAGGAPVELEPGGGRGNLPRPNMKRPPPDSFDE

SRHPRQRAAADTDAPSSGGGAGRCRDLSRAFGRCRALLDRLLRDEDGWIFAAPVDARALR

LRDYYTVIADPMDLGTVLRRLERRRYADPPAFAADVRLTFSNAKSYNNPGDPVYESADEL

SGIFEDGWASIQAELPPPPPTDAERKLKFRDDLKGLPVAAQRTVAGFLKERGACQLEKRG

KLEVDLGKADAATLDELDRVVAKHRAADSDVVAPSPECRNHEHGQTEGPSPKS*

>SiHAT19

MKRKRGRKPAGKKAAPASAAAESPSPDTPSSPSTEATNSTPDQAEEDSAPPATQAPVPAP

EPPQPAAVVAPPPHSEPPQKPAAAAAPVNPPVDIPYAKPKVGAVYGRVKLKFKSSKVVDP

PPPQQQGSSGAQAPAADAAKSETATVPEVAKEAEAEKAAILTDKQQADGQGSETSDADKE

KVVRRVGGIKIKSVGLASIGNNTPDRKADPVDEPPPSKQEAVSENKGTEETVEPRSSQEL

EEKQSTPERQRDEKELSAALEAIKKVMKMDAAEPFNIPVDPVALGIPDYFDIIDTPMDFG

TICKNLERGDKYMNSEDVYKDVQFIWDNCTKYNSKGDYIIELMKRVKKGFMKNWLAAGLY

SDVPDSGGNDNTGDEDAKGHSKSKSKNKRRRPGNDRHKSDCICAVCQVTRRKKERDEILA

VVDNETAAMDSNTSDQHDMEGNSGANHPGSHDTSSSHEQPPRTDVYKETVEADDSGIRME

DVGKFSSDRPSSLPHPDYDDEGSRQHFEGKEQVEYRDMNSNGEHTSTQPNEYSDVENHQH

KAQIETSQEVEMEEDYPMLQLNPAFQQLCASLFPSNPSSTFGVRHSLFRPRRRAPMKESP

LHAAMAELMKRR*

>SiHAT20

MMGKTHRFSKGHLLGFVPDYRHGVETVGESKGLGSPSRIDSGSSCAPPKRKCVSVNSEEG

EGASGFNVRREVFSLARMTALDKKDLEMKLRDELAQVRALQNRLFPRGPAVNMNGGVVLA

PGGGDVHPKKKVEKLKRSNSVQSDRGALPPVVAAPPVASTINYATSFKQCGNLLKSLMSH

AWAGPFLVPVDVVKLNIPDYFDIVKQPMDLGTIQKKMNAGMYSTPREFAADVRLTFSNAM

NYNPVNNDVHLMAKTLNKTFETRWRLIEKKLPQPGEKPPQPDEKPPQPDEKPPMREPSKK

NKTKRDAIEKEDPIKKKPSKKSTPKQDIFQEEDLLDNPVLQPKKRKTSPLVSSPLLQDAP

LVEAVVPTGKRIMSSEEKYELSARLQSYGALIPDHVVEFIRSHADDCGADEEELELDMDA

LGDDTLFELQKLLDDYDRVNPSRNLTEEDPHEVESRSQHELINPSVCNEEGNELIDEDID

IGENDPPVSTLPPVVFEVETADRSSKRSTSSSSSSDSESSSSDSDSSSSSGSDTDAKALP

QNSGLKESVLPVDCLDQEKGSLNTSNLPEQSTEPVSVTADGEGENVSEKQVSPEKQIRAA

LLRSRFADTILKAHEKALDQVTKKDPEKLRREREELERLQREERARLQAEAKAAEDARKK

AEAAAAAEAAAEAKRQRELEREAARKALQEMEKTVDINEGSLFLKDLEMLGSVTGEQMPN

SVGEMSPADMPEALGFQLGGNPLEKLGLYMKNDEEDDDGDFTDEPAVDVEEGEID*

>SiHAT21

MATAAAAAAVAAPEQPRRRKPAPGRGGVVLPAGLSEEEARVRAIAEIVSAMGELSRRGED

VDLNELKSAACRKYGLARAPKLVEMIAAVPEADRAALLPRLRAKPVRTASGIAVVAVMSK

PHRCPHIATTGNICVYCPGGPDSDFEYSTQSYTGYEPTSMRAIRARYNPYVQARSRIDQL

KRLGHSVDKVEFILMGGTFMSLPADYRDYFIRNLHDALSGHTSANVEEAVCYSEHSAVKC

IGMTIETRPDYCLGPHLRQMLSYGCTRLEIGVQSTYEDVARDTNRGHTVAAVADCFCLAK

DAGFKVVAHMMPDLPNVGVERDLESFREFFESPAFRADGLKIYPTLVIRGTGLYELWKTG

RYRNYPPELLVDIVARILSMVPPWTRVYRVQRDIPMPLVTSGVEKGNLRELALARMEDLG

LKCRDVRTREAGIQDIHHKIRPDEVELVRRDYAANEGWETFLSYEDTRQDILIGLLRLRK

CGRNVTCPELVGRCSIVRELHVYGTAVPVHGRDADKLQHQGYGTLLMEEAERIARREHRS

KKLAVISGVGTRHYYRKLGYELEGPYMVKCFA*

>SiHAT22

MMTQACKKRRAVYISSESGDSGTDSEVEGSKLSQKSGVTSISTCEHQSSYKIKVKSMKTS

KTRLCGNILRKLMEHKSGWLFNKPVDPVLYGIPDYFDVIRHPMDLGTVKKKLTNKQYVST

DEFAADVRLTFSNAMKYNPPGNDVHSIAKELNGIFDSEWESVERKLGGRNPVQEQQTMKV

VKIRASMDSKSRAAMDSKSTVARGTTACSARGPVACSDSLAKKTLTDAISSKVKIKFSVR

SSEQTSSKDIPVQAPGSKEGSLNHSLPTGYREASLNRSLPSTKENAKISRIQATEHSSGL

VGNESRSCNDTSTSPCASSGQGEESYLHDEPLSPSRALRAAMLRSRFAGTIVKAQQKALL

DHGKNIDPVKLQLEKERLEKRQQEEKARIEAQVKAAEAAAQRKLEEEIRMKREQEREAAR

LALRMMKKTVDIDNSDFLKELENFSKTCQSNPPGKLIVEFVDGDLPPGLGSPLERLGLFM

KQDFEDEVEQEIEDSVSPSMDIDVKKDSEEEVGRGMDDSLSPSTVIGTKDDFQEEAGHEM

EDSLSPLAVVDIKKDSDEVVEHEMVDSVSPLMDVDTEEGEISC*

>SiHAT23

MDGLAAPSPSHSGATSGGGASHRKRKLPPSSLSDATADEDDDTTAPSSPSTAPSSPSHPS

SPSSSHSDDDDDDSLHTFTAARLDGAPGGGSASGRPPKPDSSSVSAAAAAAAAAAGTGPK

PEPGSAAASDGKEDPKGLFTDNLQTSGAYSAREEGLKREEDSGRLKFLCYSNDGVDEHMI

WLVGLKNIFARQLPNMPKEYIVRLVMDRTHKSMMVIRNNIVVGGITYRPYASQRFGEIAF

CAITADEQVKGYGTRLMNHLKQHARDADGLTHFLTYADNNAVGYFVKQGFTKEITLDKER

WQGYIKDYDGGILMECKIDQKLPYVDLATMIRRQRQAIDEKIRELSNCHIVYSGIDFQKK

EAGIPRRLMKPEDIPGLREAGWTPDQWGHSKSRSAFSPDYNTYRQQLTSLMRILLKSMNE

HPDAWPFKEPVDSRDVPDYYDIIKDPIDLRTMSRRVESEQYYVTLEMFVADMKRMFNNAR

TYNSPDTIYYKCATRLENFFSGKIASQLAQASTKS*

>SiHAT11

MGSMEPSTAPENGSAAGGGGAACNGAGAAPNGGGMERRLRSSAASASWAAHLPLEVGTRV

MCRWRDQKLHPVKVIERRKGSSSSSPADYEYYVHYTEFNRRLDEWVKLEQLDLDTVETDV

DEKVEDKATSLKMTRHQKRKIDETHVEQGHEELDAASLREHEEFTKVKNIAKIELGRYEI

DTWYFSPFPPEYNDCPKLFFCEFCLNFVKRKEQLQRHMKKCDLKHPPGDEIYRSGTLSMF

EVDGKKNKVYGQNLCYLAKLFLDHKTLYYDVDLFLFYVLCECDDRGCHMVGYFSKEKHSE

ESYNLACILTLPPYQRKGYGKFLIAFSYELSKKEGKVGTPERPLSDLGLLSYRGYWTRVL

LEILKKHKGNISIKELSDMTAIKADDILSTLQSLDLIQYRKGQHVICADPKVLDRHLKAA

GRGGLDVDVSKLIWTPYKEQG*

>SiHAT2

MNVGQAAHLSGQMSGQAAQMNQVANSGVGVGVGGADGLQQHQPMQDMASLTGTDQQFVML

RTTMRDRIFECLGRKQLSAEWRKRLPELAKRLEEMLFRKFPNRNEYYNMMNGPVEPQLQF

AIKTLSAQNQQNQQNPQMSRQIASSSGYGTMIPTPGMTQGTSGNSRVPYVSDNNTLSSSG

AGMVPQNANMGTSMPGSMSNGYQHSNNTITQNSTPNSIQSAMGSVGVQRQLPHMIPTPGF

SNQQNVPVNPEYSNGTGYFNGDSAVAPHMQHQKQFSSNQNSNQIQHIGGHSNSGIHSSML

DNSSAYGLSDGHMNGGIGLHGSNMQLTNRTTAPEAYMNIPPYGSSPKPLQQQFNQRSQQR

IPTSVDMAGSGSFYATGSTPLTTANSQSMNVANLQSRSRMNPMLVNNQLNIQSIQPQPQI

KTEVLDQPEKVNFQSSQLTHDQLIRQHSMPQHQVQPNSQFVQNQYHISQQQPNPQHQQAM

LRSNSFKQSQMASSHSMQLSEQGTLPHTELVSSQASDPVDIPSFQGQYQQRNALDNVKGG

QMFGHLSGSQNFHASASHGSQQLLPSNPQLDDGSNDVSYVLKGSQTEQILRPQWQPQTTE

KAPVTTNSSLEKQIHEDFCQSTMAQDGVQQPFSSDWRLSHCTVTSIDPAVPKPLAGGFEQ

VTGNIHYLRQIRWLLLLFHAKSCTYPVGSCKFHGCVQVQELLKHFQNCQRKDCSYRSCSR

SKMVSHHYKTCVDEQCPVCSVVRKFLRQSTEQAAKQKALESRKLAQQNVTQRIMNGVEGD

RMDVDPVSAEVFDDQPSVPKRLKMQPPSPSAPANDISLTSNPHVNPGFVLQETQPELEHS

NRATYPKREVDIKADMRAPQKPIKIGYGIDGNVTTRHNMISGVPNEMNSHIKQENLSIDK

ETSETALEVKNETDDPADATVSKSGKPKIKGVSLTELFTPEQIKEHIDSLRLWVGQSKAK

AEKNQAIGHSENENSCQLCRVEKLTFEPPPIYCSPCGARIKRNAPYYTVGTGDTRHYFCI

PCYNESRGETIEVEGQAFLKAKLEKKRNDEETEEWWVQCDKCECWQHQICALFNGRRNDG

GQAEYTCPNCYVEEVKCGLRKPLPQSAVLGAKDLPRTVLSDHIEDRLFKRLKQEKQDRAA

AAGKNIDEIPGAEGLVVRVVSSVDKKLEVKSRFLEIFSKDNYPKEFPYKSKAVLLFQKIE

GVEVCLFGMYVQEFGAECSFPNQRRVYLSYLDSVKYFRPEIKTVSGEALRTFVYHEILIG

YLEYCKQRGFTSCYIWACPPLKGEDYILYCHPEIQKTPKSDKLREWYLAMLRKASKEEIV

VELTNLYDHFFITMGECKAKVTAARLPYFDGDYWPGAAEDMITQLLQEEDDRKLQKKSKT

KKIITKRALKAAGHTDLSGSASKDAMLMQKLGETIYPMKEDFIMVHLQYSCSHCCILMVS

GRRWVCHQCRSFYICDKCYDAEQQLEDRERHPSNSRDTHMLHPVDIVGVPKETKDRDDIL

ESEFFDTRQAFLSLCQGNHYQYDTLRRAKHSSMMVLYHLHNPTAPAFVTTCNVCCHDIET

GQGWRCEVCPDFDVCNACFQKGAVNHPHPLTNHPSAADRDAQNAEARQMRVQQLRKMLDL

LVHASTCRSGSCQYPNCRKVKGLFRHGMQCKTRASGGCVLCKKMWYMLQLHARACKDADC

NVPRCRDLKEHLRRLQQQSDSRRRAAVNEMMRQRAAEVAANE*

>SiHAT4

MMAQTLQGKVMASYAPSSFSIQQQMSPSDFEMLQPDNMDQSTSPIRGIIIQKIASCLKKR

EQFAKTSPDYLLKFSRKIDECLYKRAPKVHYMDLSTLEVRVNALLNSVSHRNHRDSWISS

AAPTTKNLQQLPGIQMIDSSVYHERVDPAFTNLPARARDMPTHTMFTSQRYLPYNHNVAA

ANFHLTERPESFRSTLVAPCVSALPKCSSGLGEIASAGFPNGHVKDNFPGHHILNDMSEF

SPGGAHPVDSPISSMSGSSSPLSAVCDPTTSSNAMIRSSVDSISKASGQKLSAGSDSTGE

GQSFQQYREYEKKLDGAWSQPVEQSVHSNSTTERHEMYQGQCQLDRCVEMKEKFWRMSDC

EDFCRETYSSLSSPSAQYQCCLMADCDPCDPEGERIERSEQTSNSTVSKPSSPVSDESYG

KRPVKRLKADVAGLVDVNQVESPKEQKPVVNGGNHACGETLHSEITELPTKSHCSSLGVI

NADTDDMLRQGSEGVHNMEIVTEKELHCVKDDIEMKDSKSVDQTASGVKLSLRRKGGASI

LYALTAEEIRDHLSSLINQHTCLGKLTSQETQNNEGLPDQNTCSLCGMEKLLFEPPPRFC

ALCFKIINSTGCYYAEVENGKDKSSICSKCHHLSSSRAKYVKRFDYAETDAETEWWVQCD

KCKAWQHQICALFNKKCEGAKAEYTCANCFLKEKDSGDIYALESSTVLGAQELPRTKLSD

HIEQRLSERLEQDRQQRASTSGKGAEEVPRVEGLTVRVVSSADRVLQVQPRFHEFFKQEK

YPGEFPYKSKAILLFQKIEGVDVCLFAMYVQEYGSDCPSPNQRHVYLAYIDSVKYFRPEI

KSASGEALRTFVYHEILIGYLDYCKKRGFVSCSIWACPSTKRDDYVLYCHPTVQKMPKSD

KLRSWYQNLIKKAVKEGVVVERNTLYDFFLQPANECKANISAACLPYCENDFWPGEAERL

LEKKDDKTSQKQETQVGRLLRVAKRDDRKGNLEDILLVHKLGERMRTMKEDFMMLCLQQF

CKHCHQPIVSGRSWVCTSCKNFHLCDKCHAEEQNTAQKDRHPATTKQKHAFERIEVESLP

ETDDGDPIMESKYFDSRIDFLKHCQDNQFQFDTLRRAKHSTMMILYYLHNSSCSACHHAV

DRCLVWRCLRCIGCTFCDPCYKQNGESLHIHELTQIDTSKKNAQDYVECLVHASRCFDPR

NCTLQVCLTLKKLFFHGVRCDIRARNWGGCQKCVFMWKILLHHSKGCNDTDCTVPRCRDI

KVYMAEKMKLPGPV*

>SiHAT5

MAAAAEISAMLNHLAASAERDPPARRLRSPTARPGLEALAGALAAGPPTDPAAARAVLSA

ARAVVSAVLPTSVEEVESIAVEIVERSLEFCLLHLEKSSYACDDFSLVNEVACFVEFILL

KGTHNKAYSLEPCVINDIIEQWTSVQVEAERLSPQEKYFCYLKGFNCSKSGDDLKRFRLA

LLPEYLRQDYVISENTQSCSVVSPNSMVPLAQHFAAVHLCCIPRLLTLVGKLCQSPALEM

VEDVNINLRLSFTQRILKLVCGLTMEFPPDASDAMMLSSVARCADSLPALFRLKFKFSNH

DRVFCVDGVGTILLQILEEFLQLMQIIVCNSDICCTVQVCVLSSMLEIFSPNIWRYERSG

TCLVPPLAYSPRVVQFVLKLLKDTKRWTRRVDRDKPDKDAFYYSFNHELNDLSCHVRSEE

VPLLKKYTCEEYLQFIFPSGEQWLNGLVHLTFFLHDEGVKSVTSEKPQLSCTKQPIVSEL

ESVASHEEEALFGNLFAEVRPAGITESVEQPTSLGSDLSNSQQGPIQLAADLICFMKASI

FSPEWSRAIYMDACRKFNTCHLDQFLSILKCQACFPDESSTGNTMSSENKLLHINAACFE

LLQMFLVCDECPASLREDLVEKVFNVDNGKYTYNHYALALVAHAMTYAANSGFSLGRKIF

VQYVTYLLEKANDTSSSSLNVSDFCASLPCAFHLEILLATFHLTTESEKADLVKIVLSSL

EKMKKPPSGVSVTGLTRWALLLSRLLLVLRHMLLYPLTHPSWLFMRMRSRLRDIQLNQGQ

SHSMNDCLPSLATVIVEEMLDDSVKKSAMASDLLPHLIDMTPTHADSYFDKAAVETLGLS

LADLGATMSQILNSWNGRSAEVADDLIVERYIFFICWNTLSVTGCHGNGSLLNDDYLKPE

LGNVNVFLAFALSISNGASSHAGIDLPALIFELLELLHSDILGSSKLESWDFPRKGAWLS

FILSLINADLQRQQVQVGAETEVDSHRKHEVQGEELFTHGNSLSIYLTKNIGRCLDTLSS

LLEVYLHTFKKAYLSFLYSGKPSLDNSYPSLLLKHSVFDKSKHHLLFEKSGSYLEMLEPI

YQLSSRIDGVTTKLGEGQENYFSLKCLLHGFPSAYPSSNSALLSCILVINEIMQTFNGYT

KVALPGDRDRVDEGIISKLLSMVMAVRSDQTFKPIHGECDNIFMSLINNRDDLAKYSDLF

VLKQLEGFLADINSNESMDSRMKEILVSTIVDLVEDLRSKREVFKFFLGDDAEGAPDEAS

RIFASEHADMSVFLDVLDCKSEQVNLKILHLLTDILRNGCCPGLKQKLQNKFIGMDVPCF

SSWLEFVILGPSVKVESTNGTAGPATRELAVDFFTNLICPSPEAVTKELQHHLFNSMLLL

LDRAFLSHDLQTAKAYFHFLVQLSSEESHFKHLFEKTLILMETMVEDKGKLHTLKFLFSF

VEAVFGDTGLNRSALKRLSSKTSGSSFGSGSLIPKQLKNSENLVIRTNQVSNPAVDCDAS

SGEEDEDDATSDGELGSIDRDEEDDGDSDRALASKVCTFTSSGSNFMEQHWYFCYTCDLT

VSKGCCSVCAKVCHRGHRVVYSRSSRFFCDCGAGGVRGSSCQCLKPRKFTGSSTVPPAAS

SFHPILAYHEDVEQVADSSSDFEDDISTDADSSMKLSVPNEFSSGLPLFLKNLDIEVKVL

EICKKLLPAILDRRELNLSKDRKVLLGGDVLVSHSSDIFQLKKAFKSGSLDLKIKADYPN

SRELKSHLANGSLAKLLLSISTRGKLAVGEGDKVAIFDVGQIIGQPTASPITADKTNVKP

LSRNIVRFEIVHLVFNPLVDHYLAVSGYEDCQVLTLNSRGEVTDRLAIELALQGAYIRRV

EWVPGSQVQLMVVTNLFVKIYDLSQDNISPMHYFTVADDIIVDATLVPSSMGKLVLLVLS

EGGLLYRLNVTLEGDVGAKILAETVLVKDAVSMHKGLSLYFSSTYRLLFVSHQDGTTFMG

CLNADSSSITELSYICEEDHDGKSKPAGLYRWRELLAGSGILTCLSKSKSNAPFAVSLGP

HELVAQNMRHSIGTNSSVVGVAAYKPLSKDKTHCLILYDDGSLHIYSHTPNGGESSTILA

AEQTKKLGSSILSSRAYTGIKPEFPLDFFEKTTCITSDVKYNSETTKSGDSESIKQRLTS

DDGYLESLTSAGFKVTISNPNPDIVMVGCRIHVGNTSASNIPSEITIFHRVIKLDEGMRS

WYDIPFTTAESLLADEEFTISVGRTFDGSSIPRIDSIEVYGRAKDEFGWKEKMDAALDME

ALGGSSTGGRSGKRPQIVQAAPIQEQVLADALRILSRIYLLCPPSCFTDMADAGMELNNL

KCRALLETIFQSDREPLLHSAACCVLQAVFPKKDMYYHVKDMMRLLGVIKSLPAITSRIG

VGGAASSWVIKEFIAQIHTVSKVALHRKSNLASFLETHGTELVDGLMQVFWGILDLDRPD

TQTINSLVVPCVEFIYSYAECLALHSNENSGVSVGPAVALLKKLLFAPYEAVQTSSSLAI

SSRFLQVPFPKQTMIANDDAPENHAKPSASAMGSSSGNAQVMIEEDPASSSVQYCCDGCS

TVPILRRRWHCNICPDFDLCETCYEILDADRLPVPHSKDHPMSAIPIELDTFGGEGNEIH

FSIDELTDSGVLHPPADRSVQTSPSSIHVFDASESADFPETIAGQTTVSISASKRAINSL

LLSHLIEELRGWMGTTAGTRAIPLMQLFYRLSSAVGGPFMDSSKPENLDLEKFVKWLMDE

ININKPFPAKTRCTFGEVSILVFMFFTLMFRNWHQPGSDSSHSKSSGSSDLTEKGPVQVP

ASTTVALPSSRGDQDKNEFASQLVRACSALRQQSFLNYLMDILQQLVHTFKSSSINGEAG

SSGSGCGSLLTVRRELPAGNFSPFFSDSYAKSHPTDLFTDYYKLLLENTFRLVYSMVRPE

KEKSAEKDRSNKVPNTKDLKLDGYQDVLCSYISNPHTSFVRRYARRLFLHLCGSKTHYYS

VRDSWQYSHEVKKLHKIVNKSGGFRNPVPYERSVKLIKCLSILCDVAAARPKNWQKFCLK

HMDLLPFLVDNFYHFSEECIIQTLKLLNLAFYSGKDANHNAQKPECADLGGSTRTSSQSS

DSKKKRKVDDGSEGSSEKSCMDMEQVVEMFNDKEGDLLKRFIDIFLLEWNSAGVRHEAKC

VLFGVWYHAKNPLRETMLTILLQKVTHLPMYGQNIVEYTDLMISLLGKVNDSSAKQNDSE

LVNKCLAPEVISCIFDTLHSQNELLANHPNSRIYNTLSCLVEFDGYYLESEPCVTCSCPD

VPYSRMKLETLKSETKFTDNRIIIKCTGSFTIQSVTMNVYDARKSKSVKVLNLYYNNRPV

TDLSELKNNWTLWKRAKSCHLTFNQTELKVEFPIPITACNFMIELDSFYENLQASSLESL

QCPRCSRSVTDKHGICSNCHENAYQCRQCRNINYENLDSFLCNECGYSKYGRFEFHFMAK

PSFSFDNMENDDDMRKGLAAIESESENAHRRYQQLMGFKKPLIKLVSSIGEQEIDSQQKD

AVQQMMVSLPGPTCKVNRKIALLGVLYGEKCKAAFDSVSKSVQTLQGLRRVLMTYLHQKS

SSDTNALPAFSIPRSPSSCYGCSTTFVTQCLELLQVLSKHANCRKQLVSSGILSELFENN

IHQGPRTARTLARAVLSSFSESDADAVQELNSLIQKKVMYCLEHHRSMDIAQSTREELLL

LSETCALVDEFWEARLRVAFQLLFSSIKVGAKHPAISEHIILPCLRIISQACTPPKSDGD

KESGLGISSLALQSKNDDTTGNTTTNNPSAKIQPDISGKVHDGSQRGQDIPLLSYSEWEG

GASYLDFVRRQYKVSQAVKGSIQKTRHDSHKPDYLVLKYGLRWKRRACRKSSKSDFSKFA

LGSWVSDLILSSCSQSIRSEICTLISLLCPSNSPRQFQLLNLLMSLLPRTLSAGESAAEY

FELLGIMIDSEASRLFLTVRGCLTSLCSLITKEVSNVESQERSLSIDISQGFILHKLVEL

LNKFLEIPNIRARFMSDRLLSEVLEAFLVIRGLVVQKTKLINDCNHLLKDLLDSLLLEST

ENKRQFIRACISGLQKHVKEKKRRTSLFILEQLCNLICPVKPEPVYLLILNKAHTQEEFI

RGSMTKNPYSSVDVGPLMRDVKNKICNQLDLIGLLEDDYGMELLVGGNIISLDLSISQVY

EQVWRKHHGQTQHSLSNANSLTAASSIRDCPPMTVTYRLQGLDGEATEPMIKELEEEREE

SQDPEIEFAIAGAVRECGGLEIILSMIQSLRDDEFRSNQEELASVLNLLKYCCKIRENRC

ALLRLGALGLLLDTARRAFSADAMEPAEGILLIVESLTMEANESDISIAQSVFTTTNEET

GAGEEARKIVLMFLERLCHPSGAKKSNKQQRNEEMVARILPYLTYGEPAAMEALIQHFEP

YLRDWTEFDQLQKQHEENPKDDSISRNASTQRSAVDNFVRVSESLKTSSCGERLKEIILE

KGITKAAVEHVKQSFASPGQTGFRTSAEWTSGLKLPSIPPILSMLKGLAKGHLPTQKCID

EEGILQLLHALEGVPGENEIGARAENLLDTLANKENNGDGFLGEKIQELRHATRDEMRRR

ALKKREMLLQGMGMRQEFSSDGGRRIVVSQPTIEGLDDVEEEEDGLACMVCREGYTLRPT

DMLGVYAFSKRVNLGATSAGSGRGDCVYTTVSHFNIIHYQCHQEAKRADAALKTPKREWD

GATLRNNETLCNCIFPLRGPSVPLGQYTRCVDQYWDQLNSLGRADGSRLRLLTYDIVLML

ARFATGASFSTDCKGGGRESNSRFLPFMVQMASHLADGSANQQRHAMAKAVTTYLSSSPS

TPESPIRLSASISGPRGSSGSSEETVQFMMVYSLLSESYESWLQHRPAFLQRGIYHAYMQ

HKHGRSTLKMSSDSSSSAVRSDEGSSSDMSENKKLFTIVQPMLVYTGLIDQLQQFFKKGK

SSGMGKSGEREESGGSLEKWEIQMNEKLSNMKEMVGLSKDLLSWLEDMTSSDDLQEAFDV

MGALTDVFSSGHATCEDFVRAAIHAGRS*

>SiHAT15

MNVSGQMSGQPVQMNLGGGRGLQQHQPLQVASRYPDMDHQFSKMRSAMHRNIVDHLMKMM

KIAENQSLGKLADRLEEVLYRLHPTKVDYYVMLKGPIEPHLQGAIKVLSRHGNQHQQMSH

QTPTPSHCGTMVPTPGSMSNGYQDPSINVLQNSMATSDSSVMCPVNTQRQVAHMIPAPGF

SGHQILPSNPVYPCGAGYLNGELNVIPQVHEQKPMPFSINQGSYPMQHVGTHVGFGVHSR

MLEDSSSNGLSGPQINGDIGFPGSNMQLSYGTVAAKEFTNIPPYGNSPEKLLQQEFICHP

PKGTPTSAFVSGNLHGTVSSTLKPIGNQMNAANTLPTSRMECALLTSQTTKQSLQPKPVI

KAEVLDQTENVIFTKSQLPYQHQQQHYFEPNRPCSQFVKGSHLGSCCDEQLSNQGALPYN

ELMYSKATEDNDKSDQMYRQYVSHNNIQITSGSQQLLSSHAKNTELISSMFQRPMSQDAT

EQHVSSDWPNAGCAMTPIDHKPPKLPTRGSERATNKYDLQTLRLIKFIHAKISPCPLGGS

CKSPICARLQEILKHSNDCQTIDCLYGYCKQSKEAIYHYNNCVNKHCPICSKAKSLSHYC

DQTNKRNTFERSINGANGDRMGINMVTAETFDDQPPMSKRLRLQLLPPNVSHSADASVPQ

ACTGIVSQQAHPKHLGQDKMIFPKQEQNIEIDIQSPRKVEIIRSCAVGKTGAIQTYVVPD

VSNELDSYIEKKNCLSDKDTNEIVVDIKNNANGSTDAMMSKIEKTKRKGVSLMELFTPEQ

IHEHVRSLRQWVGQSKAKAEKNQVIGHSKNVNSCQLCKVEKLFFEPPPKYCSPCGARIKR

NAPYYSDTVTESGPYYFCIPCYSESRSDSILVDNIQLLKSKLVKNRNDDELEEAWVACDK

CKRWQHQICALFNAKRNDEEKDAEYICHSCYIQEIEHGLRMPLPLNTVPGAKDLPRTVLS

DHIEERLLQRLKEERQNRANKYGKNFSEVPGAEGLVVRVVSSVDKKLKVKPHFLEIFRED

NYPAEFPYKSKAILLFQRIEGVEVCLFGIYVQEFGAECAFPNQRRVYLSYLDSVKYFRPE

IETVSGEALRTFVYHEILIGYLQYCKQRGFTSCYIWACPPFKGEDYIMYCHPEIQKTPKS

DKLREWYLSMLRKATNEGIVVELTNLYEHFFNPKTECKAKVTAARLPYFDGDYWPGAAED

IINQIRLPEDDRNLQKKGKLKKTITKRALKFAGLTNLNGNASKDAMLMQKLGEAIYPMKE

DLIMVHLQHSCHHCCILIVSGRRWVCSQCKSFYICDKCYNVEEQREAKERHPSNSTDFHI

LHPVEIDGVPKDTKDRDGILESEFFDTRQAFLSLCQGNHYQYDTLRGAKHSSMMVLYHLH

NPTEPAFVTTCDVCKNNIKTGQGWRCKECDYDECVACYKHNEGANHVHKLTKQPTGADMD

THQKKSAETTQMLLRLLAHAGSCPGRGGCQYHNCRKLKSLFHHGTQCKTRSSGGCRLCKK

LWGLITLHARGCKESQCNIPRCRDTKEYWRKLQLMQWQSESRRRAAVNQMMMQRQLETSS

RAAGNGVNV*

>SiHAT17

MECLELDSSDFFLDGDPIDSPLEIHLESNSLASTKAVPDHRRYAQLHCSNAPDPPPLPGT

SYGARRSSRKTTACSRVPGGILDSWDKLFLEGYQADLRVSTDDGSEILSHSCVLGVKSPV

LRAMLEDAELTNGFRCIRISGAPSGAVHVFIRFLYSSRFEQEQIKKHVLHLLVLSHVFSV

PSLKTVCIDQLERNFLAPDNVVDILQLAGLCDAPRLSLICTRMIIGDFKTISLTDGWKVM

RRVNPNLEQELLESLVEADTKRQERAKKMEEKKVYLQLYEAMEALIHICREGCRTIGPRD

QALKGSRAGVCKFPACKGIELLVRHFSGCSVRVPGGCANCKRMWQLLELHSRMCFSPDTC

KVPLCRHFKEKMQHLGRKEETKWNLLVCKVLESRGAMSFISERRKFSALKTAGPGSRHVA

PCLVGPI*

>SiHAT18

MTQTLQGKVMASNAPSSFSIRQQMSPSDFDMLQPDNMDQSTSPIRSIVIQKIASCLGKRE

LFAKFSPDYLLKISRNIDELLYRSAPKVRYMDLDTLEARVDALLSSVSYRNHRDSWVSSA

AASTKNLHQLPGIQMTDSSVYHDVVAPGFTNLPARARDVPTHTMFTSQRYLPHNYNVVAA

NFPLTVRPESFRTTIVSPCVSVPPKCSSGLGGTASDGLPNGHVKDHFPGDAHPVDSPISS

MSGSSSPLSAVCDPTTSPSAMIRSSMDSVSKASGQKLSAGSDVYSTGEGQSFQQHREYER

ELDGAWSQPVELSIQSNRTTERHELYLKGECHLDRCMEVEGKYCRVSDCEDLCREKYSSL

STPRAQYQCCFMTDCDPCDPERERVGRSEQTSNSTVSKPSSTVSDESYGKRPAKRLKADV

PSLVNVNQAESPKEQKPVVNENHAYGETVQSEITELPTKSPCSSSGDINADTNNTLEQGS

EDVHNMDVVAEEELHCVKGDIEMKDSKTVALDQTANQVNISSRRKRGASILYALTAEELR

DHLSSLINQHTCLSKVISQEIQLIEGLPDQNTCSFCGMERLLFEPPPRFCALCFKIINST

GCYYAEVENGKDKTSICSKCHHLSSSRAKYVKRFNYAETDAEAEWWVQCDKCKAWQHEIC

ALFNRKCEGAKAEYTCAKCFLKEKDSGDIHALESSTVLGARELPRTKLSDHIEQRLSERL

EQDRQQRANASAKGAEEVPRVEGLAVRVVSSADRVLQVQPRFHDFFKQEKYPGEFPYKSK

AILLFQKIEGVDVCLFAMYVQEYGSDCPSPNQRHVYLAYIDSVKYFRPEIKSASGEALRT

FVYHEILIGYLDYCKKRGFVSCSIWTCPSTKRNLEDILLVHKLGERMRTMKEDFIMLCLE

QFCKHCHQPIVSGRSWVCTSCKNFHLCDRCHAEEQNTAQKDRHPATTKQKHAFQRIEVEP

LPETDDGDPTMESKYFDSRIDFLKHCQDNQFQFDTIRRAKHSTMMILYYLHNSTCSACHC

AVDQCLVWRCLECLGCTFCDPCYKQNGESLHIHELRQIDASKTMQMNAIQDYVEGLVHAS

RCFDPCNCTLQVCLTLKKLFFHGVRCDIRARNWGGCNKCVFMWKLLLWHSKHCNDANCMV

PRCRDIKAYMTEKIKLGGPVL*

>SiHAT7

MVAMEGKGDASVTPVRTSDRLRQRPKYYARGYMYYKPAMRKKVKSKKRTAASQIAKKLLR

KPAARPPPADSIAANLRRSTRKRRIPVNLEGYDTDSSSMEDDDLMRPRYRTSKNKGGNDA

AHDEVSARPKRQKLSNSIPRREGLRPRRSLRGRRLHPYQESEDEQESSEEQGAEDRRENG

NDIEEDGDDDEVDGGDEAEADGDDEDGEEEQEGRRRYDLRDRSEVRRPSPRKEGKHRPQS

PRRVLVHGIGPKNNKYLKKGGSRMHKRPRFSLPDDSDDSLLVDEPDEGPSMPWMRSGRGG

MPWLMGGLDMHSPAAWGLNVGASGWGHQGDSSTSLMPGVQTAGPSSKGGADIQPLQVDES

VSFKDIGGLSEYIDALKEMVFFPLLYPDFFANYHITPPRGVLLCGPPGTGKTLIARALAC

AASKAGQKVSFYMRKGADVLSKWVGEAERQLKLLFEEAQKNQPSIIFFDEIDGLAPVRSS

KQEQIHNSIVSTLLALMDGLDSRGQVVLIGATNRIDAIDGALRRPGRFDREFYFPLPGYE

ARAEILDIHTRKWKDPPPKELKMELAASCVGYCGADLKALCTEAAIRAFREKYPQVYTSD

DKFVIDVDSVSVEKYHFLEAMSTITPAAHRGSIVHSRPLSSVIAPCLKRHLDKIMERISD

IFPFLSSVDVSKFSALSYGSSIPLVYRPRLLICGGESVGLDHVGPAVLHELEKFSVHSLG

LPSLLSDPSAKTPEEALVHIFGEAKRTTPSILYLPQFHLWWDTAHEQLRAVLLTLLNELP

SNLPVLLLGTSSVAFTDLEEECASIFTSRNVYQVDQPSYDDRLRYFNILFESLLSFQTEE

SRNKSKKQKSAIDLPKAPKEVEGPKISELKAKAEAEQHAVRRMRMCLRDICNRILYNKRF

NVFHFPVSEEEVPDYRSVIHKPMDMATVLQRVDSGQYLTRAAFMKDIDLIVLNAKTYNGD

DYNGSRIVSRACELRDVVQGMLSQMDPSLVSFCDKIASQGGPLQVVDDEDSSILQAAPVA

QLVSGTRISARLRNVQPEVNLSQSYEVLKRQKKSSENDQGMTKDAAARDERSPEDVDLSK

PISPEEAPKEPDSNGTLKETDNSPAEAPEVPAPPEPMETDSSEVATTLTTGDDLLGQLEA

LKQRFMELTAGYGVPQLERLYSRIMKGAIELTSKESNEDHRGLVVRYLLTFVENSDNF*

>SiHAT8

MGKQQQAPPSRRPPAMSAPPPPRRRKKKGRPSLLDLQKRSLRLEQQLQEQKQQPQARRST

SRNPDDEDDDDGPASGSGRREKKLRLVMGLHDGSAKGQKTRKATDGREEPSDSGPTTPLP

NKKLLLFILDRLQKKDTYGVFSEPVDPEELPDYHDIIKHPMDFSTIRKKLDKGAYSNLEQ

FEDDVFLISSNAMCYNSPDTIYYRQARGIQEIAKKDFENLRQDSDASEPEPEPEPEPEPE

PEPEEPKPQPRRGRPPNKNNARQKVGRPPAERATADFSGATLANAANSGRHAQPDLDLSR

RAMDKAMIADVLRASFANRRNEHNWSGERKSERIEDYSGYGSMWSAKMGKKPILMEDSRR

STYYEAQPSSSIYELPVSSSYNGTKKLLVPVGVQLQQSYSRSLARFAAQLGPIGWEIASR

RIERSLPPETKFGPGWVGDGEAPSSFQPPALAAFSETMAPPSNVAASGDQPPNNSGPAAE

DCAASSSHLAVSQPHAMPYPSTSTVQRTDSGALASQQRGSIPHIPINRGEHDAEMKGGHN

NLHGHPAMQQPVNGFNAVPGPVMFPPAAQLVVNQMQTHTAD*

>SiHAT10

MPRSPSPSPSPSPERHHHQPARRRVGATSSQMLHHHGGRSPSPPPRRSLRPRRAAAVSSR

PLVDDFFPFPSSPSSSPSRPRQRRPSPEPSSSDSGADGGGGGSSASDRRRRKLKLVVKLS

QLPPEQQHRRAPPPPSYSDDSDGAGEVGGDGSDDDEQVKPPKKRRIEPRADRSRHREVGG

GGRSDPASAPRTKRLPVPGTARTTPLPDRKALEMILEKLQKKDTYGVFAEPVDLEELPDY

HDVIEHPMDFGTVRRKLARNAYRSFEQFEDDVFLICSNAMQYNAPDTIYFRQAHSIQELA

RKKFQELRDEGIPIENHHIKIEQKARPNSCNREPIKKPILRYQDDDLDFLSRKEQVKRSN

PKNSEDDISFKDQVKKPVPRNLHDESSLFHKERVKKPISRNSENDLSSSFHKDRPKKLLS

RSSEDDLSSSFGKEQVRKVIPKNSENDESITFHKQQVKKTTSQSSKSDFSSQKKHIKKPV

CSTREGPDLSSRKEPVEDPICTNVDDAGFLSTKRLEEKPISRNSEDLGHCHQESPKKPSC

RDGQDDLGNSCSEEAAKKPARMNSQDAMGSDISAATIASVGDGSNGLSMSQANATEPAGC

TVANGFLDKDISSPLDEIRSEKTDDIFAKPNYKSIVVDETRRKTYDTYEEQPAVESDLVF

DIFSTEPKELVNVGLDAEHSYAYARSLARFAGSLGAQGWRIASERIRQALPAEVKYGRGW

VGEYEPPLPSILVVNDQSRYLKSSEANVRRNASLPRDNDRLRPTESNNPKDMSLSLNRIT

TSTNVVGVPGPLESPEFKPRLFGVTAEPQHRSTDALSPHENHRVPGNVAKTKRTANEQTR

KGNSSSGARPIEMKPQKGASGAPDMPALNKTAGQPRPFFQPAESTRTQQMRKVDSLKSNV

PIEMAPQRLECAKGAASGVYDTPSSNGQPKHFFPSQAAAASGVHDIPSPNGQPKRLFQSK

AVASSGAHDAPSNGQPKHFFQSQGPASSGVHDMPSTNGQPKPFFQPQEATVPQPRNEATW

VYHGRPGDGKVGTSNKSRPSTSVGFVNKNQAVNAATFAMNLNGQKNVSDHAKSVGSTAMP

GLANIPNRGVDASRNMFSAFPAAVRENQSIPPAPSAQSWISFGAATENKPAIVSPTFLDN

NSSWKMPFANVRPSDDTKIGAVPQFFRQPVQVVRESPVQNNGLVIFPQLVQPDFMRSQGQ

PQWQGLVPHMQQKPSKDVLRPDLNIGFPSPGSPPARQSSGINLEAQQPDLALQL*

>SiHAT12

MRDNESPTVSAADDDEEEDYEEPGGGNGFLGFMFGNVDDSGDLDADYLDEDVKEHLFALT

DDLGQSLEDIDLIRSSPAPTDPSEQDYDDKAEDAVDFEDIDEEYNGPEVEATTEEENVLS

RKDYFSSNAVYALVNSTVSVFDEENYDEDDETTNDIEIHVNSVAQNCSSDVLTEQPVMEP

SNIMNFEYEVLQSILTQKEMGTEEGHLGSETAVSLPVLCIEDGSVILRFCEIFDIQEPAR

KRKADHHTHPINKELRIAKYADIIEEDEEVFFRSSIHNSSNLKHIKMDEDFGESDSDESV

PDVTLCLNDSCRSEQPMKDSHQDIPTAKQSPVCPDFYALEHDDWENYIIWDDSPPATESQ

PFLKSCVIYEESMDTHCEDRAKDFGHPTGCCDVKSKIHVSPVIIQPFGFTKMPAASNYHA

PENSYRALTKETAQDKNNHTEPNRIAGTLKTKTMQCLDNLYSLNRELLEGSWWDNIIWDP

CEDTLKPRLIFDLKDDRMLFEILDEKKVDLIHSHAPAMSVGSQSGQSSTSSVEKFDNQSI

SWSDHFNISNDEFYSNWKWSQQAKSSSKKGASIHIKVVHSAPAQKLQTMKLKLSNKEIVN

FHRPKAKWYPHENKLAAQLQGVASSHGRMTAILMTLGRKGFKLVFNADETPVSVKLKVSK

KLEFKPSERIKLFCSGKELQDDISLAMQNVYPNSILHVVRSEVNLWPKAQKLPGEGKPLH

PPRAFRKKADLSVKDGHVFLMEYCEERPLLLSNAGMGARLCTYYQKTSPADETATSLQKN

SNGLGTVLAIDPADIPPFLGDIHSGSHQSCLETNMYRSPIFPHQVSSTDYLLVRSTKGVL

SLRRIDKLYAVGQQEPHMEVLSPGTKTVQNYLLDRMLVYVYREFRARERPGVISQIRADE

LPIQSPLTDAMVRKRLKHCAELKKGPYGHSFWTQRPDFQVPSEEELRRLLAPESVCCYDS

MQAGLYRLKKLGIVNLTHPVGLASAMNQLPDEVMELSAAANIERELQITSWNLTSNFVAC

TNEDRKNIERLEITGFGDPSGCGLGFSYVKKKSAPAKGTLVTGTDADLRRLSNDAARELL

LKFGLPEEQIDKLTRWDRITMVRKLSSEKAISGITINEIPVSKFARRNGMSFMQLQQQTR

EKCQEIWDRQVESLSAVDHVENGSDTEANSDLDSFAGDLENLLDAEEFDDEDTGKAGLRN

DKAEGMRGLIMRRCPTRTQINGEIEDDVEEVSLAKKLLEDDGNDTKGKKQPVDMTNYGTS

IYGRGAIKSKQSETGQMIKSYAHSAALTPKGSTATEVQEARNSFAEGRLPLKLKAAMTFD

GNDILLVKRSALGMDALEEKRQCGKYGTLICGACGQLGHIRTNKLCPKYGEDQENSGMDT

NSTKSNPLDEVSQMRIKTPSKKLTTMVSSEVPETRGPECIEKKKSVQVKFKCGDDYPPDT

IKASVVFRPPAELGKDIPCKMITIKQPKVLVDQERHVEFLASENKSREDWCDRESGQMNS

LHGSRSSLEERSSSNRIIMENDGSLITFKGKRDIQEQMPIETRIHEKREKGLRKAKQKIM

EKRKPESGGDALLDHRPYINERRVPEKHRASKRRRGGEVELSNILEKVVDQLRRNTAISY

LFLKPVMKKDAPDYFDIVKRPMDLATIRDKVRKMEYRNRQSFRLDVAQIAVNAHAYNDNR

HRGIPPLADELLKMCDQLLEESAELLDDAEGAIED*

>SiHAT14

MSDGERRDEENPTASAADDDDDEDYEEPGGGNHFLGFMFGNVDDSGDLDADYLDEDAKEH

LFALADKLGPSFKDIDLIKSSPAPTDPSEQDYDEKAEDAVDYEDIDEEYDGPEVEAATEE

DNVLSKKDYLSSAVYASVNNTVSVFDEENYDEDEETPNDNKSTGDNAVQNLSSVSSEQAD

MATSSDHLPLEKTGSLSYPEESMDFEYEVLENEMGTEEGQLEPETVTSLPVLCIEEGNVI

LRFSEIFGIQEPVRKVKTDHHKRPVNKELQISNVADSVEEDEEIILRSTTIQNFSTLKHI

QMNEDFVETDSDESVADVTLRLKDSCLSEQPMKVAHTVQRSPVCPDFYPLEHDDWENDII

WNNSPSNDCQPYAKICESEESVDTHGEDQAKDYGQASRCWDVQSKSNGSPVIEEPFGCTE

MPAPANYHSPGNNHPPLTNEDNIDHIMPNNLDEAVKTDTMLRLNHLSLLNRELLEGSWLD

NIIWDPSEGTPKPKLIFDLKDDHMLFEILDEKNVDHLRSHARAMIVSQSVKTSTTTVENF

DSQVKALSGRFNISNDKFYSNRKAPQQAKSHTKKRALMGIKVVHSAPAHKLQTMKPVLSN

KEIANFHRPKAKWYPHENKIAAQLQGAACSHGRMTAILMTLGGKGVRILVNSEDTPVSVK

LKASKKLELKPSEKIKLFCSGKELQDDISLAMQNVRPNSILHVVRTEVTLWPKAQKLPGE

DKPLRPPGAFRKKTDLSVKDGHVFLMEYCEERPLLLSNAGMGARLCTYYQKTSPADQTAS

SLRNNGDGLGTVLAIDPADKSPFLGDIRSGSHQSCLETNMFRAPIFPHKVAPTDYLLVRS

AKGVLSLRRIDKLYAVGQQEPHMEVFSPGTKNVQNYLLNRVLAYVYREFRARERPDGIPQ

IRADELPIQSPLTEAIVKKRLKHCADFKKGPKGHFFWTQRPDFRVPSEEELRRLLTPESV

CCYESMQAGLYRLKRLGIVKLTQPVGLASAMNQLPDEAIELAAASHIERELQITSWNLTS

NFVACTNQDRENIERLEITGVGDPSGRGLGFSYVRVAPKAPSSNSMLKKKSAAAKGTTVT

GTDADLRRLSMDAARELLLKFGVPEEQIDKLTRWHRIAMDVFLQLQQQTREKCQEIWDRQ

LQSLSAIDGDDNGSDTEANSDLDSFAGDLENLLDAEEFDDEDTSTAELRSDKADGMRGLK

MRRCPTHAQINEEIEDDEAEASLAKKLLEDNGNDMKRKKQPEGLTNCGTSTGANKTKQNK

TGQMIKSSGYAGASTPKESTPREAKEAENSFAEGGLPTKLKTKVAFDGNDILLVKKKSAL

GKDGPKEKRQGARGDTLVCGACGQLGHMRTNKLCPKYREDQETSEMDANSVKSNPTDIVN

HLPTKTPKRLITKVSSEATETEGPECIEKTKSVPVKFKLGAPDKSLERNMSLSSSLVSEK

RTMDVTDYRSTGKVNKIIIPNRIKSDDYPPDTPKPSVVFRPPAEEKDVPRKKITIKQPKG

VDQQKLVEPRSGQEPTRKTRKMVELSSFEGKSREDDHWGLGLEGKRRSKAVMESEKSWRD

FEEQREMPQQRLTDARIYASREEDHQKAKKKNKKKKKHEFRDDDLLDHRPYKNDRRVPER

QRAVKRSIPVDMIEHTPSAKRRRGGEVELSNILEKIVDDLRGNTQISLLFLKPVTRKDAP

DYLEIIQRPMDLGTIRDKVRKMEYRNRYEFRHDVAQIQLNAHIYNDERHPHIPPLADALM

EMCDYLLDESADELAEAEDAIEH*

>SiHAT24

MGPHTAPPHRSGNGGPRKSRAARRKPKGPRKKIPNARKATRTPTRSQAITPPPPPPPRPR

LAAPPPPLPSPPSRACRTPMAKTRKPPPPPPPPPPPPPPPAETPSPQRRRKKKGRPSLLD

LQRRSLRLQAQNPSPAPSPSRRDPNPSDDDEDGAGSGRRRQKRLKSVLSGVVKEEPGEGK

KDAAKATGKGLAALDGGGPTGTPLPDKKLLLFILDRLQKKDTYGVFSEPVDPEELPDYHE

IIEHPMDFSTIREKLLNDSYSNLEQFEDDVFLLTSNAMSYNSADTIYFRQARSIEALAKK

DFENLRQPSDEEEEPKPPARRGRPPKNPKTEDDVSPDLSNVKTNKPEDNADTIRKRSTGD

RTRNTTPLKDPSTFHSMFGSFSAKRTDKIGDYSGSSKWGKKPPSLDDDRRSTYDQHYSRN

SSLFGAFDDERKLLVPVGVQQQHAYARSLARFAAKLGPVGWDIAANRIRRALPPGTNFGP

GWIVDGEPPQNSQWPPVVTSTNPSESTAPPNMPSKIDVLHHKSGPSSNGAVTGEEHLTRT

QTVPSTSASFDKSSEIASKVIKHENGANKSCGGIDNTGPAPPLQHHSHSREIHSNINGFS

AVSNAMSQFAGHGLFGSGIPMTHAQVLGMFSGMNGKANGYIHGHQLTADSLKAAQNGDVG

KATVNPVQGASHDPKIVNDNSSAHPSLNAGVQSSGSLPKGKLVNPKHPDLALQL*

>AtHAG1

MDSHSSHLNAANRSRSSQTPSPSHSASASVTSSLHKRKLAATTAANAAASEDHAPPSSSF

PPSSFSADTRDGALTSNDELESISARGADTDSDPDESEDIVVDDDEDEFAPEQDQDSSIR

TFTAARLDSSSGVNGSSRNTKLKTESSTVKLESSDGGKDGGSSVVGTGVSGTVGGSSISG

LVPKDESVKVLAENFQTSGAYIAREEALKREEQAGRLKFVCYSNDSIDEHMMCLIGLKNI

FARQLPNMPKEYIVRLLMDRKHKSVMVLRGNLVVGGITYRPYHSQKFGEIAFCAITADEQ

VKGYGTRLMNHLKQHARDVDGLTHFLTYADNNAVGYFVKQGFTKEIYLEKDVWHGFIKDY

DGGLLMECKIDPKLPYTDLSSMIRQQRKAIDERIRELSNCQNVYPKIEFLKNEAGIPRKI

IKVEEIRGLREAGWTPDQWGHTRFKLFNGSADMVTNQKQLNALMRALLKTMQDHADAWPF

KEPVDSRDVPDYYDIIKDPIDLKVIAKRVESEQYYVTLDMFVADARRMFNNCRTYNSPDT

IYYKCATRLETHFHSKVQAGLQSGAKSQ*

>AtHAG3

MATAVVMNGELKKQPRPGKGGYQGRGLTEEEARVRAISEIVSTMIERSHRNENVDLNAIK

TAACRKYGLARAPKLVEMIAALPDSERETLLPKLRAKPVRTASGIAVVAVMSKPHRCPHI

ATTGNICVYCPGGPDSDFEYSTQSYTGYEPTSMRAIRARYNPYVQARSRIDQLKRLGHSV

DKVEFILMGGTFMSLPAEYRDFFIRNLHDALSGHTSANVEEAVAYSEHSATKCIGMTIET

RPDYCLGPHLRQMLIYGCTRLEIGVQSTYEDVARDTNRGHTVAAVADCFCLAKDAGFKVV

AHMMPDLPNVGVERDMESFKEFFESPSFRADGLKIYPTLVIRGTGLYELWKTGRYRNYPP

EQLVDIVARILSMVPPWTRVYRVQRDIPMPLVTSGVEKGNLRELALARMDDLGLKCRDVR

TREAGIQDIHHKIKPEQVELVRRDYTANEGWETFLSYEDTRQDILVGLLRLRKCGKNVTC

PELMGKCSVVRELHVYGTAVPVHGRDADKLQHQGYGTLLMEEAERIARREHRSNKIGVIS

GVGTRHYYRKLGYELEGPYMVKHLL*

>AtHAG2

MVQKQQASAGPGTEPKKRRRVGFSPADTGVEANECIKIYLVSSKEEVDSSDISSVKPVDL

NDFFDGDGKIYGYQGLKINVWINSISLHSYADITYQSTINGDKGITDLKSALQNIFAETI

VDTKDEFLQTFSTQRDFIRNMVSNGEVMHAGATDGSSKNAEVVPSDPQVIRMEIGSPNAG

LLYSRLVPLVLLFVDGSNPIDVTDPDWHLYLLIQKKEEKEDPLYRIVGFTAIYKFYRYPD

RLRMRLSQILVLPSFQGKGLGSYLMEVVNNVAITENVYDLTVEEPSEKFQHIRTCIDINR

LRSFDPIKPDIDSAVQTLTKGKLSKKAQIPRFTPPLNAIEKVRESLKINKKQFLKCWEIL

IYLALDPIDKYMEDYTSVITNHVRTDILGKDIETPKKQVVDVPSSFEPEASFVVFKSVNG

EEANTNVQVDENKPDQEQQLKQLVEERIREIKLVAEKVSKSGQTLKV*

>AtHAM1

MGSSADTETAMIIATPASNHNNPATNGGDANQNHTSGAILALTNSESDASKKRRMGVLPL

EVGTRVMCQWRDGKYHPVKVIERRKNYNGGHNDYEYYVHYTEFNRRLDEWIKLEQLDLDS

VECALDEKVEDKVTSLKMTRHQKRKIDETHVEGHEELDAASLREHEEFTKVKNIATIELG

KYEIETWYFSPFPPEYNDCVKLFFCEFCLSFMKRKEQLQRHMRKCDLKHPPGDEIYRSST

LSMFEVDGKKNKVYAQNLCYLAKLFLDHKTLYYDVDLFLFYILCECDDRGCHMVGYFSKE

KHSEEAYNLACILTLPPYQRKGYGKFLIAFSYELSKKEGKVGTPERPLSDLGLVSYRGYW

TRILLDILKKHKGNISIKELSDMTAIKAEDILSTLQSLELIQYRKGQHVICADPKVLDRH

LKAAGRGGLDVDVSKMIWTPYKEQS*

>AtHAM2

MGSSANTETNGNAPPPSSNQKPPATNGVDGSHPPPPPLTPDQAIIESDPSKKRKMGMLPL

EVGTRVMCRWRDGKHHPVKVIERRRIHNGGQNDYEYYVHYTEFNRRLDEWTQLDQLDLDS

VECAVDEKVEDKVTSLKMTRHQKRKIDETHIEGHEELDAASLREHEEFTKVKNISTIELG

KYEIETWYFSPFPPEYNDCVKLFFCEFCLNFMKRKEQLQRHMRKCDLKHPPGDEIYRSGT

LSMFEVDGKKNKVYAQNLCYLAKLFLDHKTLYYDVDLFLFYVLCECDDRGCHMVGYFSKE

KHSEEAYNLACILTLPSYQRKGYGKFLIAFSYELSKKEGKVGTPERPLSDLGLLSYRGYW

TRVLLEILKKHKGNISIKELSDVTAIKAEDILSTLQSLELIQYRKGQHVICADPKVLDRH

LKAAGRGGLDVDASKLIWTPYKDQS*

>AtHAC1

MNVQAHMSGQVSNQGTMSQQNGNSQMQNLVGGGSAPATGAGLGPSRVSPVDNDILKLRQA

MRIRIFNILQQKQPSPADEASKAKYMDVARRLEEGLFKIANTKEDYVNPSTLEPRLASLI

KGRQLNNYNQRHANSSSVGTMIPTPGLQHSGGNPNLMITSSGDATMAGSNNITTSAMNTG

NLLNSGGMLGGNLSNGYQHSSSNFGLGSGGNMSSMSSQRNTGQMMPTPGFVNSSTNNNSN

NGQSYLSVEASNNSGGFSTAPMMVPQTQQQQLRQDIGGQNSRMLQNHGSQMGVGLRPGMQ

QKLSNVSNSSINGGVGMNAKSVDSGTSYTNPIRNSQQAYDNLQRSGMQGDGYGTNNSDPF

GSGNLYGAVTSVGMMTNTQNANTASFQAVSRTSSSLSHQQQQFQQQPNRFQQQPNQFHQQ

QQQFLHQQQLKQQSQQQQRFISHDAFGQNNVASDMVTHVKHEPGMENPSESIHSQTPEQF

QLSQFQNQYQNNAEDRHAGSQILPVTSQSDMCTSVPQNSQQIQQMLHPHSMASDSVNGFS

NLSVGVKTESGMRGHWQSQSQEHTQMSNSMSNERHIQEDFRQRMSGTDEAQPNNMSGGSI

IGQNRVSTTSESLNPQNPTATTCRNGNGNRDPRFKNQQKWLLFLRHARHCKAPEGKCPDR

NCVTVQKLWKHMDSCAAPQCSYPRCLPTKTLINHHRSCKEPNCPVCIPVKAYLQQQANAR

SLARLKNETDAARSVNGGGISSDAVQTSAGAKSCTSPGADISGHLQPSLKRLKVEQSSQP

VDVETESCKSSVVSVTEAQSSQYAERKDHKHSDVRAPSKYFEVKAEVSDFSVQTRPGFKD

TKIGIAENIPKQRPVSQPDKQDLSDVSPMQETTKVEKEPESLKKENLAESTEHTSKSGKP

EIKGVSLTELFTPEQVREHIRGLRQWVGQSKAKAEKNQAMEHSMSENSCQLCAVEKLTFE

PPPIYCTPCGARIKRNAMYYTVGAGDTRHYFCIPCYNESRGDTILAEGTPMPKARLEKKK

NDEETEEWWVQCDKCEAWQHQICALFNGRRNDGGQAEYTCPYCFIAEVEQSKRKPLPQSA

VLGAKDLPRTILSDHIEQRLFKRLKQERTERARAQGKSYDEIPTAESLVIRVVSSVDKKL

EVKPRFLEIFREDSYPTEFAYKSKVVLLFQKIEGVEVCLFGMYVQEFGSECAFPNQRRVY

LSYLDSVKYFRPEVRSYNGEALRTFVYHEILIGYLEYCKLRGFTSCYIWACPPLKGEDYI

LYCHPEIQKTPKSDKLREWYLAMLRKASKEGIVAETINLYDHFFMQTGECRAKVTAARLP

YFDGDYWPGAAEDLIYQMSQEEDGRKGNKKGMLKKTITKRALKASGQTDLSGNASKDLLL

MHRLGETIHPMKEDFIMVHLQPSCTHCCILMVSGNRWVCSQCKHFQICDKCYEAEQRRED

RERHPVNFKDKHALYPVEIMDIPADTRDKDEILESEFFDTRQAFLSLCQGNHYQYDTLRR

AKHSSMMVLYHLHNPTAPAFVTTCNACHLDIETGQGWRCEVCPDYDVCNACFSRDGGVNH

PHKLTNHPSLADQNAQNKEARQLRVLQLRKMLDLLVHASQCRSAHCQYPNCRKVKGLFRH

GINCKVRASGGCVLCKKMWYLLQLHARACKESECHVPRCRDLKEHLRRLQQQSDSRRRAA

VMEMMRQRAAEVAGGSG*

>AtHAC2

MAPPRKRTRDLMPKFLNTESFDEFNQRLNNLPAESNVTSDEDAQFLESRKCQSKRWRKEE

PLKLNLRSPWNVLCSPESISSAKFIVEKTCLIPVPSFEEAATNARRCLNTSSIPGSSGSA

SETNSGSDITKQDFKNDSPSDSKKVQGSSTSKSAKPKVIKVYSFVDLVTTTKKGNIQTEE

SSLNHEKKLGTVVDIVEPMKCDERSKEVQGSSTSKSAKPKVIKVYSFADVVTTTKKGNIQ

TEESSLNHEKKLGTVVDIIEPMKCDERSKEVQGSSTSKSAKLKVIKVYSFADVVTTTKKG

NIQTEESSLNHEKKLGTVVDIVEPMKCDERSKEVQGSSTSKSEKPKVIKVYSFADVVTTT

KKGNIQTEESSLNHEKKLGTVVDIVEPMKCDEGTKCEVTTTNKGKIHTEERSLNHEKKLG

TVVDIVEPMKCDEGSKCEVTTTNKGNTQTEERSLNHEKKLGIGVDIVEPMKCDEGTKCEV

TTTNKGKIQTEERSLNYEKKLGIGVDIVEPMKCDEENKCEVNADTFDVVIVEPMKCNKVT

KCEVNVDTTGVNIVEPMKCNEVTKCEVNVDTIGVDIVEPMKCNEESKCEVNADTMSLQKR

SKRAVSLVERFTEEEIKLHIMSLKKPSTQSAVEGMCDLKEEEESCQLCDDGTLLFPPQPL

YCLLCSRRIDDRSFYYTPGEEELSNAQHQICSPCHSRCKTKFPLCGVFIDKHKMLKRSNF

DNADTEEWVQCESCEKWQHQICGLYNKLKDEDKTAEYICPTCLLEECQSINNMALVDYTD

SGAKDLPETVLSYFLEQRLFKRLKEERYQTAKATGKSINDVPEPEGLTLRVVFSADRTLT

VNKQFASLLHKENFPSEFPYRSKVILLFQKVHGVDICIFALFVQEFGSECSQPNQRSTYI

FYLDSVKYFKPERVTFAGEALRTFVYHEVLIGYLEYCKLRGFTTSYIWACPPKIGQDYIM

YSHPKTQQTPDTKKLRKWYVSMLQKAAEQRVVMNVTNLYDRFFDSTEEYMTAARLPYFEG

SFWSNRAEIMIQDIEREGNNELQKKVKLLSRRKVKTMSYKTTGDVDVDDVKNILLMEKLE

KEVFPNKKDLMVVELNYSCTRCSKAVLSGLRWFCEKCKNLHLCESCYDAGQELPGEHIYK

RMDKEKHQLSKVQVNGVLFSTTEDNDIIQENDMFESRQAFLAFSQKHNYNFHTLRHAKHS

SMMILHHLHTSNKHHCSQNSSSLTCTACKKDVSTTIYFPCLLCPDYRACTGCYTKNRTLR

HLHIFPTLPSANRAPSRTVMVLEILNAISHALLCQHKTTKSCSYPKCHEVKALFTHNVQC

KIRKKGTRCNTCYKLWQTIRIHVYHCQDLNCPVPQCRDRKEVLIRKV*

>AtHAC5

MAQGQNRTVLQSGQMHNSVVASASSVSSQPNMVNGISQDTLLLRQEMLNRTYAWLQQRQP

SKTDDASKAKLSEVAKRLESAMWRTATSKEDYLDFRSFDVRVESTLKQLLSQRRANPSSS

VSTMVQTPGVSHGWGQSYTATPMVDTSKFNSSNNLSDATTETGRLLPTNRMYRGTINNGR

QELSAVGQMIPTPGFDNSANADVYQSHRNEEYSGDGGKLLATGSDFGNPSQLQKQRPTGS

NDLMGYNLDHQLGGGFRSNIHQNTSGMTSIPLNAGVGMSGNNVHLANVPRSSEGVLSSTH

FSTFSQPSQQPVEQLQVSHVNRYSMSNSGTFVSGNLYGVQTSSGSIETAVDMNSMSLNSM

RRVDTSFGSQSGLQNNPLLKPHLRHQFENGNFQSSSNSKENLAQVSHRPLERQFNQQAHY

GQYHQQELLMNNDAYRQSQPASNLVSQVKNEPRVEYYNEAFQMQAINKVEPSKPQNQYKQ

NTVKDEYVGAQSAPVSSSQLKMSPSFPPQTHQTQQVSQWKDSSSLSAGVQPVSGLGQWHS

SSQNLTPISKNSNEEREHFGVRFHKQHEGTNNSSSVRESTNCLTVAPSGTLDVPHLPVGI

NVLSKQLNGDCGLSYKNQRRWLLFLLHVRKCNAAEDNCESKYCFTAKTLLKHINCCKAPA

CAYQYCHQTRQLIHHYKHCGDEACPVCVFVKNFKEKQKEKFTFLQRAEPSSASLNHGPKE

SFESMRTSSERDSEAPFVVDDLQPSPKRQKVEKPSQFAYPDTQGNPATISAGVSQAHFSM

GLQEKDRLPSDVCKPVRSNVPMNADSSDSSRRLVPVSRELEKPVCKDTHMGRHGVKSALD

GESLRLSKQEKPKRMNEISAPKEENAEQSLGVVSASNCGKSKIKGVSLIELFTPEQVEEH

IRGLRQWVGQSKTKAEKNKAMGLSMSENSCQLCAVERLAFEPTPIYCTPCGARVKRNAMH

YTVVAGESRHYVCIPCYNEARANTVSVDGTPVPKSRFEKKKNDEEVEESWVQCDKCQAWQ

HQICALFNGRRNHGQAEYTCPNCYIQEVEQGERKPVSQNVILGAKSLPASTLSNHLEQRL

FKKLKQERQERARLQGKSYEEVPGADSLVIRVVASVDKILEVKPRFLDIFREDNYSSEFP

YKSKAILLFQKIEGVEVCLFGMYVQEFGTDSASPNQRRVYLSYLDSVKYFRPDVRTVSGE

ALRTFVYHEILIGYLDYCKKRGFSSCYIWACPPLKGEDYILYCHPEIQKTPKTDKLREWY

LAMLKKASKEKVVVECTNFYDHFFVQSGECRAKVTAARLPYFDGDYWPGAAEDLIDQMSQ

EEDGKKSNRKLMPKKVISKRALKAVGQLDLSVNASKDLLLMHKLGEIILPMKEDFIMVHL

QHCCKHCCTLMVSGNRWVCNQCKNFQICDKCHEVEENRVEKEKHPVNQKEKHVLYPVAID

NIPTEIKDNDDILESEFFDTRQAFLSLCQGNHYQYDTLRRAKHSSMMILYHLHNPTVPAF

AMACAICQQELETAQGWRCEVCPDYDVCNACYSKGINHPHSIISRPSATDSVVQNTQTNQ

IQNAQLREVLLHVMTCCTAQCQYPRCRVIKGLIRHGLVCKTRGCIACKKMWSLFRLHSRN

CRDPQCKVPKCRELRAHFSRKQQQADSRRRAAVMEMVRQRAADTTASTPE*

>AtHAC12

MNVQAHMSGQRSGQVPNQGTVPQNNGNSQMQNLVGSNGAATAVTGAGAATGSGTGVRPSR

NIVGAMDHDIMKLRQYMQTLVFNMLQQRQPSPADAASKAKYMDVARRLEEGLFKMAVTKE

DYMNRSTLESRITSLIKGRQINNYNQRHANSSSVGTMIPTPGLSQTAGNPNLMVTSSVDA

TIVGNTNITSTALNTGNPLIAGGMHGGNMSNGYQHSSRNFSLGSGGSMTSMGAQRSTAQM

IPTPGFVNSVTNNNSGGFSAEPTIVPQSQQQQQRQHTGGQNSHMLSNHMAAGVRPDMQSK

PSGAANSSVNGDVGANEKIVDSGSSYTNASKKLQQGNFSLLSFCPDDLISGQHIESTFHI

SGEGYSTTNPDPFDGAITSAGTGTKAHNINTASFQPVSRVNSSLSHQQQFQQPPNRFQQQ

PNQIQQQQQQFLNQRKLKQQTPQQHRLISNDGLGKTQVDSDMVTKVKCEPGMENKSQAPQ

SQASERFQLSQLQNQYQNSGEDCQADAQLLPVESQSDICTSLPQNSQQIQQMMHPQNIGS

DSSNSFSNLAVGVKSESSPQGQWPSKSQENTLMSNAISSGKHIQEDFRQRITGMDEAQPN

NLTEGSVIGQNHTSTISESHNLQNSIGTTCRYGNVSHDPKFKNQQRWLLFLRHARSCKPP

GGRCQDQNCVTVQKLWSHMDNCADPQCLYPRCRHTKALIGHYKNCKDPRCPVCVPVKTYQ

QQANVRALARLKNESSAVGSVNRSVVSNDSLSANAGAVSGTPRCADTLDNLQPSLKRLKV

EQSFQPVVPKTESCKSSIVSTTEADLSQDAERKDHRPLKSETMEVKVEIPDNSVQAGFGI

KETKSEPFENVPKPKPVSEPGKHGLSGDSPKQENIKMKKEPGWPKKEPGCPKKEELVESP

ELTSKSRKPKIKGVSLTELFTPEQVREHIRGLRQWVGQSKAKAEKNQAMENSMSENSCQL

CAVEKLTFEPPPIYCTPCGARIKRNAMYYTVGGGETRHYFCIPCYNESRGDTILAEGTSM

PKAKLEKKKNDEEIEESWVQCDKCQAWQHQICALFNGRRNDGGQAEYTCPYCYVIDVEQN

ERKPLLQSAVLGAKDLPRTILSDHIEQRLFKRLKQERTERARVQGTSYDEIPTVESLVVR

VVSSVDKKLEVKSRFLEIFREDNFPTEFPYKSKVVLLFQKIEGVEVCLFGMYVQEFGSEC

SNPNQRRVYLSYLDSVKYFRPDIKSANGEALRTFVYHEILIGYLEYCKLRGFTSCYIWAC

PPLKGEDYILYCHPEIQKTPKSDKLREWYLAMLRKAAKEGIVAETTNLYDHFFLQTGECR

AKVTAARLPYFDGDYWPGAAEDIISQMSQEDDGRKGNKKGILKKPITKRALKASGQSDFS

GNASKDLLLMHKLGETIHPMKEDFIMVHLQHSCTHCCTLMVTGNRWVCSQCKDFQLCDGC

YEAEQKREDRERHPVNQKDKHNIFPVEIADIPTDTKDRDEILESEFFDTRQAFLSLCQGN

HYQYDTLRRAKHSSMMVLYHLHNPTAPAFVTTCNVCHLDIESGLGWRCEVCPDYDVCNAC

YKKEGCINHPHKLTTHPSLADQNAQNKEARQLRVLQLRKMLDLLVHASQCRSPVCLYPNC

RKVKGLFRHGLRCKVRASGGCVLCKKMWYLLQLHARACKESECDVPRCGDLKEHLRRLQQ

QSDSRRRAAVMEMMRQRAAEVAGTSG*

>AtHAC4

MNNNKEVPQNSVAVSSSSSAPITVISPQQDANNFIKKRRTALRNRIYAIVRHKQKQHQIF

LDKKNQQQQQRVDDATQRALLEKQDQQCIAATRMIEEELLKSSRSFEEYFDLRTFDARVR

TILQQLGTMLSQRRAAAAAMNNGEAQCITSTRAVHTSISVSNSFQCGRSLVPINCTTATA

GAFSIGPDMQTHHSTGANHQMVEVNRPNMNQITCGISSPLITGFNGNCVPVSANIPMTSQ

DLFNATHFSTLPQPFLQPPPDQSHMHRYSMSNVASFGQSNPYPCGVVMSSGSMAVAQNSI

PWNNPNPMQGLDPTVTSYHSNLQPMQQTPLPKRQLHHPLWNTNFQSAPNNRDNLPQVSQQ

LSNHGSRQHRGQHSQNLYPGQLQNQDRLLPNLTQQAMALAAPVMHVPSKQVNEDCGQTSS

NTVLRWIPFMFHARHCKAKKDKCASKFCFQARKIVKHIDCCKVPNCKYRYCLGTRMWLDH

FKQCKSISCRTCVAVREYMEKNKYTIVPLRRAKCSSASSKCQPKKSSKSRQAYKKGGAEA

PSVDADLQRSIKRPKLHRPSQNITPETKSISVTGCGVVCKPHSLMNMQEKDGLQSLKVEA

MPMDIDVPGASEIPVTRELVKHVAEDTPKGNNCGGFAMVEKTSCLLAQGKSKCMNEMSAP

KEENVKQSVEVVDASKMEISSLVELFTPEQVKEHIRSLRQWVGQSKTKAEKNKAMGCSMS

VNSCQLCAVEWLVFEPVPIYCSPCGIRIKKNALHYSIAVGESRHYVCAPCYNEAREKLVF

LDGTSIPKTRLQKKKNDEQVPEGWVQCDKCEAWQHIICALFNSRRNHGESTKYTCPSCYI

QEVEQRERRPLPLSAVPGATSLPVTSLSKHLEERLFKKLKEERQERARLQGKTYEEVPGA

ESLTVRVVASVDKVLEVKERFLELFREENYPSEFPYKSKAIFLFQKIENVEVCLFGMFVQ

EFGTDSGPPNERRVYLSYLDSVKYFRPTFRTVSGEALRTFVYHEILIGYLDYCKKRGFTS

CYIWACPPLKGDDYILYCHPEIQKTPKTDKLREWYLAMLRKASKEDVVVECTNLYNHFFV

QSGECRANVTAARLPYFDGDYWPSAAEDLLRQMNQEDDGETKLHRKGLTKKVISKRALKA

VGQLDLSLNASKDRLMMQKLGETICPMKEDFIMVHLQHCCKHCTTLMVSGNRWVCNHCKN

FQICDKCYEVEQNRINIERHPINQKEKHALFPVAIKDVPTKIEDKDNNLESEFFHNRQAF

LNLCQGNNYQYETLRRAKHSSMMILYHLHNPTAPAFATVCTICQQEVENSQGWHCEVCPG

YDVCSACYSKDSINHSHKLTSRSSSTDSTVVQQNGQASQSYQVKLEKLKKLLVHAATCRS

TQCQYQGCRKSKMLFRHCIDCTTGDCPICKGLWSLLKLHARNCRDSKCTVPKCSGLRAIS

RRKQQQADKRRRAAVMEMMRERAAEATRTG*

>AtTAF2

MICRVDYGSNDEEYDGPELQVVTEEDHLLPKREYLSAAFALSGLNSRASVFDDEDYDEQG

GQEKEHVPVEKSFDSEEREPVVLKEEKPVKHEKEASILGNKNQMDTGDVQEELVVGLSEA

TLDEKRVTPLPTLYLEDDGMVILQFSEIFAIQEPQKKRQKREIRCITYRDKYISMDISEL

IEDDEEVLLKSHGRIDTHGKKTDQIQLDVPLPIRERSQLVKSGIVRDTTSESREFTKLGR

DSCIMGELLKQDLKDDNSSLCQSQLTMEVFPLDQQEWEHLILWEISPQFSANCCEGFKSG

LESAGIMVQVRASNSVTEQESLNVMNSGGQTQGDNNNMLEPFFVNPLESFGSRGSQSTNE

STNKSRHHPQLLRLESQWDEDHYRENGDAGRENLKQLNSDARGRLSGLALQDRDMWDESW

LDSIIWESDKDLSRSKLIFDLQDEQMIFEVPNNKERKYLQLHAGSRIVSRSSKSKDGSFQ

EGCGSNSGWQFNISNDKFYMNGKSAQKLQGNAKKSTVHSLRVFHSAPAIKLQTMKIKLSN

KERANFHRPKALWYPHDNELAIKQQKILPTQGSMTIVVKSLGGKGSLLTVGREESVSSLK

AKASRKLDFKETEAVKMFYMGKELEDEKSLAEQNVQPNSLVHLLRTKVHLWPWAQKLPGE

NKSLRPPGAFKKKSDLSNQDGHVFLMEYCEERPLMLSNAGMGANLCTYYQKSSPEDQHGN

LLRNQSDTLGSVIILEHGNKSPFLGEVHGGCSQSSVETNMYKAPVFPHRLQSTDYLLVRS

AKGKLSLRRINKIVAVGQQEPRMEIMSPASKNLHAYLVNRMMAYVYREFKHRDRIAADEL

SFSFSNISDATVRKYMQVCSDLERDANGKACWSKKRKFDKIPLGLNTLVAPEDVCSYESM

LAGLFRLKHLGITRFTLPASISTALAQLPDERIAAASHIARELQITPWNLSSSFVTCATQ

GRENIERLEITGVGDPSGRGLGFSYVRVAPKSSAASEHKKKKAAACRGVPTVTGTDADPR

RLSMEAAREVLLKFNVPDEIIAKQTQRHRTAMIRKISSEQAASGGKVGPTTVGMFSRSQR

MSFLQLQQQAREMCHEIWDRQRLSLSACDDDGNESENEANSDLDSFVGDLEDLLDAEDGG

EGEESNKSMNEKLDGVKGLKMRRWPSQVEKDEEIEDEAAEYVELCRLLMQDENDKKKKKL

KDVGEGIGSFPPPRSNFEPFIDKKYIATEPDASFLIVNESTVKHTKNVDKATSKSPKDKQ

VKEIGTPICQMKKILKENQKVFMGKKTARANFVCGACGQHGHMKTNKHCPKYRRNTESQP

ESMDMKKSTGKPSSSDLSGEVWLTPIDNKKPAPKSATKISVNEATKVGDSTSKTPGSSDV

AAVSEIDSGTKLTSRKLKISSKAKPKASKVESDSPFHSLMPAYSRERGESELHNPSVSGQ

LLPSTETDQAASSRYTTSVPQPSLSIDKDQAESCRPHRVIWPPTGKEHSQKKLVIKRLKE

ITDHDSGSLEETPQFESRKTKRMAELADFQRQQRLRLSENFLDWGPKDDRKWRKEQDIST

ELHREGKVRRAYDDSTVSEERSEIAESRRYREVIRSEREEEKRRKAKQKKKLQRGILENY

PPRRNDGISSESGQNINSLCVSDFERNRTEYAPQPKRRKKGQVGLANILESIVDTLRVKE

VNVSYLFLKPVTKKEAPNYLEIVKCPMDLSTIRDKVRRMEYRDRQQFRHDVWQIKFNAHL

YNDGRNLSIPPLADELLVKCDRLLDEYRDELKEAEKGIVDSSDSLR*

>AtTAF1

MAESNGKGSHNETSSDDDDEYEDNSRGFNLGFIFGNVDNSGDLDADYLDEDAKEHLSALA

DKLGSSLPDINLLAKSERTASDPAEQDYDRKAEDAVDYEDIDEEYDGPEVQVVSEEDHLL

PKKEYFSTAVALGSLKSRASVFDDEDYDEEEEQEEEQAPVEKSLETEKREPVVLKEDKAL

EYEEEASILDKEDHMDTEDVQEEEVDELLEGTLDDKGATPLPTLYVEDGMVILQFSEIFA

IHEPPQKRDRRENRYVTCRDKYKSMDISELVEDDEEVLLKSHGRIDTHVEQADLIQLDVP

FPIREGLQLVKASTIGGITPESREFTKLGRDSCIMGELLKQDFIDDNSSLCQSQLSMQVF

PLDQHEWERRIIWEHSPEISGNSGEIFEPGLEPEGMLVKGTNSETEQESLNVVNSRVQVQ

ADNNMFVPFSANLLESFGSRGSQSTNESTNKSRHHPQLLRLESQWDENHLSGNDEAGVKK

IKRLEKDALGRFSRLVLRERDLGDEAWLDSIIWDSEKELSRSKLIFDLQDEQMVFEIFDN

EESKNLQLHAGAMIVSRSSKSKDETFQEGCESNSGWQFNLSNDKFYMNGKSSQQLQANTN

KSSVHSLRVFHSVPAIKLQTMKSKLSNKDIANFHRPKALWYPHDNELAIKQQGKLPTRGS

MKIIVKSLGGKGSKLHVGIEESVSSLRAKASRKLDFKETEAVKMFYKGKELDDEKSLAAQ

NVQPNSLVHLIRTKVHLWPWAQKLPGENKSLRPPGAFKKKSDLSTKDGHVFLMEYCEERP

LMLSNAGMGANLCTYYQKSSPEDQRGNLLRNQSDTLGNVMILEPGDKSPFLGEIHAGCSQ

SSVETNMYKAPIFPQRLQSTDYLLVRSPKGKLSLRRIDKIVVVGQQEPRMEVMSPGSKNL

QTYLVNRMLVYVYREFFKRGGGEHPIAADELSFLFSNLTDAIIKKNMKIIACWKRDKNGQ

SYWTKKDSLLEPPESELKKLVAPEHVCSYESMLAGLYRLKHLGITRFTLPASISNALAQL

PDEAIALAAASHIERELQITPWNLSSNFVACTNQDRANIERLEITGVGDPSGRGLGFSYV

RAAPKAPAAAGHMKKKAAAGRGAPTVTGTDADLRRLSMEAAREVLIKFNVPDEIIAKQTR

WHRIAMIRKLSSEQAASGVKVDPTTIGKYARGQRMSFLQMQQQAREKCQEIWDRQLLSLS

AFDGDENESENEANSDLDSFAGDLENLLDAEEGGEGEESNISKNDKLDGVKGLKMRRRPS

QVETDEEIEDEATEYAELCRLLMQDEDQKKKKKKMKGVGEGMGSYPPPRPNIALQSGEPV

RKANAMDKKPIAIQPDASFLVNESTIKDNRNVDSIIKTPKGKQVKENSNSLGQLKKVKIL

NENLKVFKEKKSARENFVCGACGQHGHMRTNKHCPRYRENTESQPEGIDMDKSAGKPSSS

EPSGLPKLKPIKNSKAAPKSAMKTSVDEALKGDKLSSKTGGLPLKFRYGIPAGDLSDKPV

SEAPGSSEQAVVSDIDTGIKSTSKISKLKISSKAKPKESKGESERRSHSLMPTFSRERGE

SESHKPSVSGQPLSSTERNQAASSRHTISIPRPSLSMDTDQAESRRPHLVIRPPTEREQP

QKKLVIKRSKEMNDHDMSSLEESPRFESRKTKRMAELAGFQRQQSFRLSENSLERRPKED

RVWWEEEEISTGRHREVRARRDYDDMSVSEEPNEIAEIRRYEEVIRSEREEEERQKAKKK

KKKKKLQPEIVEGYLEDYPPRKNDRRLSERGRNVRSRYVSDFERDGAEYAPQPKRRKKGE

VGLANILERIVDTLRLKEEVSRLFLKPVSKKEAPDYLDIVENPMDLSTIRDKVRKIEYRN

REQFRHDVWQIKYNAHLYNDGRNPGIPPLADQLLEICDYLLDDYEDQLKEAEKGIDPND*

>OsHAC701

MMAKTLQGTQQQYAASGFPTQQYPTSGWTQSAAEILQLDNMDQDTSVVRNIIHRKIVEYL

NERKEFCNFDLSFLMEIGKCIDRHLFEKADSKIKYMDLETLRTRLNAIVNSASFRGSMFH

WSASAASSKLNSQQLPVMEVPIYHDRVTPGPNNLPSCAYNVSSTQGYNQYENCMGAANFA

HSLADKPKQMPERLANTIFTSCASTLPKCSPSIDVLHIGHIKEHFSGDAYQNDSSQPSTS

GSSSSLSAVWDQTTCSSAMRTLPMDSFSTVNGQNLSTNNKSLYPTTGQGPLLQQYIECEM

KQETWSRSLEQSDQSNITTGNRDLYHAQIHPYINGEHKRDRCIQMKEKLGHTSDHEGFSR

EKSSNLSNHFMHHQQGFMTNYGACSPVSKTVDRAEQTSNSTVSKPTSPASDGSSGKHYPA

KRLKVDVPHLVHVNEMEASKEQQPAANETYASAETVQSEVTNSPTKSPCCTSLGDNIACT

DNVHGMDMVRLSGSAVQTEEEFRRENSDIEMKDAKVDLLDQTLSGDSLRARKRRGASVLY

ALTSEELKDHLCTLNHDTSQSKVPTEELLSVEGLPDQNTCNLCGMERLLFEPPPRFCALC

FKIINSTGSYYVEVENGNDKSSICGRCHHLSSAKAKYQKRFSYAETDAEAEWWVQCDKCK

AWQHQICALFNPKIVDPEAEYTCAKCFLKEKDNEDVDSLEPSTILGARELPRTRLSDHIE

QRLSERLVQERQQRAIASGKSVDEVPGVEGLTVRVVSSADRTLQVQPRFKDFFKKEQYPG

EFPYKSKAILLFQKNEGVDVCLFAMYVQEYGSACPSPNQRHVYLAYIDSVKYFRPEIKSA

SGEALRTFVYHEILIGYLDFCKKRGFVSCSIWTCPSTKRDDYVLYCHPTIQKMPKSDKLR

SWYQNLVKKAVKEGVVVERNTLYDFFLQPTNECKTNISAAWLPYCDNDFWPGEAERLLEK

KDDDTSQKKETQLGRLLRVAKRDDRKGNLEDILLVHKLGERLRTMKEDFLMLCLQQFCKH

CHHPIVSGSSWVCTSCKNFFLCERCYAEELNTPLKDRHPATTKQKHAFERIEEEPLPETD

DVDPTMESKYFDSRIDFLKHCQDNQYQFDTLRRAKHSTMMILYHLHDSTCSSCHRAMDQC

LAWRCLVCLGCNFCDSCYKQDGESLHIHKLRQKKDHHVLQKYTLQDYLEGLVHASRCFDR

SCTSKLCLTLKKLFFHGVRCHTRARGGGGCHMCVFMWKLLFTHSLLCDNADCSAPRCRDI

KAYIADRSMTDLSISG*

>OsHAC703

MNVGQAAHLSGQMSGQAPQTNQVGGSGVGGADGLPQQMQDVVGLGGLDTQFLLMRNTMRD

RIFEYIGRKQSSTDWRRRLPELAKRLEEILYRKFLNKADYLNMMRGPVEPQLQFAIKTLS

AQNQQNQQNQQMPRQMASSSGYGTMIPTPGITQSATGNSRMPYVTDNTGLPSSGATMVPQ

GANTGSMSNGYQHLTTSVPLNSTTSSIPSTMGPVGIQRQVTHMIPTPGFNNQQNVPVNPD

FSNGAGYFNGEPTVTSQMQQQKQFPSNQNSHQIQHIGGHSNSGMHSNMLENSSAYGLSDG

HVNGGMGVHGSNMQLTNRSAASEAYINISTYGNSPKPVQQQFNQHPPQRIPTPVDISGSG

NFYNTGSSALTAANNHSMGATNLPSRSRMNSMLHTNQLNMQSIQPQPQIKTEVLDQPEKM

NFQSSQLTHEQLIRQQHSMQQHQMQPSSQFVQNQYHLNQQQPNSQHQQSILRSNSLKQPQ

LSSSHSMQLSEQGALPHTELISSQATEHADIPIYQGQYQQRSAHDNVKGGQVFGHLSSSQ

NFHSNASHDSQQLLPTNQQLDDSSNDVSYVLKGSQPEQMHQAQWRPQTMEKAPVTNDSSL

EKQIQADLCQRTMSQDGAQQPFSSDWRLPGCTVTPADPALPKLPSGGLEQAAGNIYYFRQ

MKWLLLLFHAKSCLTPVGSCKFHRCFQVQELVKHFENCKRKDCSYRDCRRSRMVTEHYKA

CVDLQCPVCSNAKKLLQRSAELASKQKPPEPRKIAQQNTAQRIMNGVEGDIMDIDLVSDE

IFDSQPSVPKRLKMQPVSPSTAEREVSMPSNAGLILQETHSELPDQNNKVGQLKMDVKID

PRPLQKPAKIGYGTDGNVPTARHNVAPGGSNEIKTHVKQEIMPIDKETSETAPEVKNEAN

DSTDITVSKSGKPKIKGVSMTELFTPEQIQEHINSLRLWVGQSKAKAEKNQLMGHNENEN

SCQLCKVEKLTFEPPPIYCSPCGARIKRNAPYYTVGTGDTRHFFCIPCYNESRGDTIEVE

GQNFLKARFEKKRNDEETEEWWVQCDKCECWQHQICALFNGRRNDGGQAEYTCPNCYVEE

VKRGLRMPLPQSAVLGAKDLPRTVLSDHIEDRLFKRLKQERQDRAAQERKSIEEVPGAEG

LVVRVVSSVDKKLEVKPRFLEIFQEDNYPTEFPYKSKAVLLFQKIEGVEVCLFGMYVQEF

GAECSYPNQRRVYLSYLDSVKYFRPEIRTVSGEALRTFVYHEILIGYLEYCKQRGFTSCY

IWACPPLKGEDYILYCHPEIQKTPKSDKLREWYLSMLRKATKEEIVVELTNLYDHFFITM

GECKAKVTASRLPYFDGDYWPGAAEDMINQLRQEEDDRKQQKKGKTKKIITKRALKAAGH

TDLSGNASKDAMLMHKLGETIYPMKEDFIMVHLQYSCSHCCTLMVSGKRWVCHQCRSFYI

CDKCYDAEQQLEDRERHPSNSRDTHTLHPVDIVGLPKDTKDRDDILESEFFDTRQAFLSL

CQGNHYQYDTLRRAKHSSMMVLYHLHNPTAPAFVTTCNVCCHDIETGQGWRCEVCPDFDL

RKMLDLLVHASTCRSGSCQYPNCRKVKGLFRHGMQCKTRASGGCVLCKKMWYMLQLHARA

CRDSGCNVPRCRDLKEHLRRLQQQSDSRRRAAVNEMMRQRAAEVAANE*

>OsHAC704

MKQGQGAHLSGQRIGHHPTAQMNPGDGDGNGRHQVASGHASADPELMNLRIRMTNRLIWE

LLSREPKLQTRPRKLVSDLAKRFEAVIYKKNPNKAAYYSILNGEIFPHLQHALSTHMAQH

QQGQQMLQQLTSSSSYGTTIPIPDVVQNASGNTRALYEMDNTSGPMSNGHHHFSANFPLH

STTKGASLEMSAVSMQEGKITHMIPTPGSSNQQSLPGNFHYSTGTGYLNGKSNVMAQMQE

QQAPFASKINCCPVQRDLGGYAGSGVHSDILNNSSPYGVSEAHMIDGMGLHRSNVQVINR

TVVPETFINPSPYGISPNKPLQRHVNPSTRSTPTPADIAASTSFNGTGSSALSTTSYLDM

TTVNSLPKSRMDSGLIMSQPTIQSFQTEYYIQTEGLDLQEKISLEQLHQQVNQLHLIQPH

SQYAQNQCSLKLQQQNSLHHLVMSRGNVLTQCHLGSDHAEKLLDKRNQLHSELVSSQINE

HVGLTNLQGHYEQTQYHDNYKKGQMSASSQNLGIPAPHDLLPPQQQFDDGSYRLSCFLKE

TYTKPLQPHCKSKPMKEVIMTSLLSGKIQDGFCQKKMARDREHHPIISGWHSAGCAATSF

GSEEVMENTKQYHAQARWLLFLFHAKSCTSPPGSCKSSYCDRVRELVVHLTDCQIKDCSY

RHCRESKMVSDHYKNCINEHCHVCCKAKEMLRRSSELAHKQNPAEPILITQHNMNQRSAD

RVHGDRMDIDQAVETFDDQPPAAKRPKLQLVSPDASENVPVCQKNPGFMLQEAHPRQLDQ

NKKMVPDQEVDVGLDIRHPQVTLVSCHGSDEKIGAAQNTVIPGALNKIHCHVQQETVVAD

KESVTVVDVKKKTGSVDVTISKTGKPKVKGVSLMELFTPEQIHEHINSLRQWIGQWVQCD

KCECWQHQICALFNARRNDVEEAEYTCFKCYIEEFKRGLRMPLPESVVRGAKDLPRTLLS

DHIEERLFKRLREERQERANKLKTSLDEVPGADGLVVRVVSSVDKKLEVKPHFFKILQED

NYPAEFPYKSKAILLFQKIEGVEVCLFGMYVQEYGAECKFPNQRRVYLSYLDSVKYFRPD

IETVSGQALRTYVYHEILIGYLEYYKQRGFTSCYIWACPPVKGEDYILYCHPEIQKTPKS

DKLRQWYLSMLQKAIKENIVVELTNLYDQFFVTAKECKIKVSAARLPYFDGDYWPGAAED

IINQLQLEGDGKLLKKGRVNKIITKRALKAAGHTDLSGNASKEAMLMQKLGEIICPIKDD

LIMVHLQYSCSHCCTFMVSGRRWVCNECKSFYICDRCYNAEQRLEEKERHPSNSKCLHIL

HPVEIVGVSEDTKDRDIILENEIFDTRQAFLSFCQGYHYQYDTLRRAKHSTMMMLYHLHN

PTGPAFVATCNVCNCDIENGQGWDFKSFERKQNQLSESRRMASVNERVRQRVAEVTRHE*

>OsHAF701

MGDGERREDENPTTSAADDDDDEDYDEPGGGNHFLGFMFGNVDDSGDLDADYLDEDAKEH

LFALADKLGPSLKDIDLIKPSAAPTDPSEQDYDAKAEDAVDYEDIDEEYDGPEVEAATEE

DHLLSKKDYFSSNAVYASVNSKVSVFDEENYDEDEEPPNDNDLPSDNIVQNCTSASAEQL

DMAPSNDNLAVEKMSSSLSEPEESFESEAFQKEMVAEEQLESKTATSLPVLCIEDGSVIL

KFSEIFGAQEPVRKAKMDRHKRPVNKELQITNFTDIVEEDEEVFLRSTIQNLSALKHIKT

NDNFVESDSDESTSDVALRLKDSCLSEQPMKDKDIPTAVQSPVFPDFYPLEHENWENDIV

WGNSPTTAIQPCLTSCAISKESLDDHNEDQAEGYVSGCWDVQNKFHSSSVMADPFGHTEI

PDSTSYRSPENSYSPLRKETAQENNSLDEPNNITQPVKIDTTRHLNKLSLLNKELLEGSW

LDNIVWDPSEDVPKPKLIFDLKDDHMLFEILDEKNGDHLRSHARAMIVTRPMKTSAVENV

DHNNQAIALSGRFNISNDKFYSNRKMSQQARSHAKKRATMGLKLVHSVPAQKLQTMKPKL

SIKEIANFHRPKAKWYPHENKLTARFQGDECSHGPMTAIVMTLGGKGVKFLVNAEETPLS

VKSKASKKLEFKPSEKIKLFCSGKELQDDISLAMQNVRPNSILHVVRTEIHLWPKAQRLP

GENKPLRPPGAFRKKSDLSVKDGHVFLMEYCEERPLLLANAGMAARLCTYYQKTSPSDQT

ATSLRSNSDGLGTMLAIDPADKSPFLGNIRSGSHQSCLETNMYRAPVFPHKVATTDYLLV

RSPKGMLSLRRIDKLYAVGQQEPHMEVFSPGTKNMQNYILNRILVYVYREFRAREKPGII

PQIRADELPIQPPITEAIVRKRLKHCADLRKGPKGHLFYIQRPDFRIPSEEELRRLLTPE

NVCCYESMQAGQYRLKHLGIEKLTQPVGLASAMNQLPDEAIELAAAAHIERELQITSWNL

TSNFVACTNQDKENIERLEITGVGDPSGRGLGFSYVRVTPKAPVSNSTHKKKSAAAKGTT

VTGTDADLRRLSMDAARELLLKFGVPEEQIDKLTRWHRIAMVRKLSSEQAASGVTMDEIP

VSKFARGQRMSFLQLQQQTKEKCQEIWDRQIQSLSAMDGNENGSDTEANSDLDSFAGDLE

NLLDAEEFDDEDVGNTDIRSDKMDGMRGLKMRRCHTQSQINEEIQDDVAEAALVEKLLEE

SDSDMKRKKQPVETTNYSTPMYNQGNKMKQGKAGQMIKSSVYAGALTPKESIPREAKEVE

NFAEGSLPSKLRTKTGFDANDDIILVKRKNIPGKDGFKEKRQGARGDTLVCGACGQLGHM

RTNKLCPKYGEDPETSEMDVNSIRSHPPDIVSNAQIKTSNKRLVAKVSSEAFETEGPESI

EKAKPVPVKFKCGAPEKSLDRNMSISASLVSDKRMMDATDSKSTGKVNKIKISNKIKYDD

YPPDTPKPSVVIRPPAEVEKDLPRKKIIIKQPKVLGDQQRPTELRSGQEPRKTRKIVELS

SFEKRDREDDNGFSGQPIQINSSHDRGWGLVGKRSKGIMESSESWRAFEEQRERQEQRLI

EARIYDARREDELQKAKKKNKKKKKHEFRDDDLLDPRPYKNDRRVPERGRAAKRRTPADM

TEYTPPAKRHRGGEVELSNILEKIVDHLRTMSCSFLFRKPVTKKEAPDYFDIIERPMDLG

TIRDKVRKMEYKNREDFRHDVAQIALNAHTYNLNRHPHIPPLADELLELCDYLLEESADV

LDDAEYAIED*

>OsHAG702

MDGLAAPSPSHSGATSGGGASHRKRKLPPSSLSDATADEDDDTTAPSSPSTSPSSPSRPS

SPSSSHSDDDDDDSLHTFTAARLDGAPPSSSGRPPKPESSTVSAAAAAAAAAAAPKPDSA

SAAAGDGKEDPKGLFTDNIQTSGAYSAREEGLKREEEAGRLKFLCYSNDGVDEHMIWLVG

LKNIFARQLPNMPKEYIVRLVMDRTHKSMMVIRNNIVVGGITYRPYTSQKFGEIAFCAIT

ADEQVKGYGTRLMNHLKQHARDADGLTHFLTYADNNAVGYFVKQGFTKEITLDKERWQGY

IKDYDGGILMECRIDQKLPYVDLATMIRRQRQAIDEKIRELSNCHIVYSGIDFQKKEAGI

PRRTMKPEDIQGLREAGWTPDQWGHSKSRSAFSPDYSTYRQQLTNLMRSLLKNMNEHPDA

WPFKEPVDSRDVPDYYDIIKDPIDLKTMSKRVESEQYYVTLEMFVADMKRMFSNAKTYNS

PDTIYYKCASRLESFFSNKVASQLAQASTKN*

>OsHAG703

MATAVAAAGGGGGGEQPRRRKPAPGRGGVVLPAGLSEEEARVRAIAEIVSAMGELSRRGE

DVDLNALKSAACRRYGLARAPKLVEMIAAVPEADRAALLPRLRAKPVRTASGIAVVAVMS

KPHRCPHIATTGNICVYCPGGPDSDFEYSTQSYTGYEPTSMRAIRARYNPYVQARSRIDQ

LKRLGHSVDKVEFILMGGTFMSLPADYRDYFIRNLHDALSGHTSANVEEAVCYSEHGAVK

CIGMTIETRPDYCLGPHLRQMLSYGCTRLEIGVQSTYEDVARDTNRGHTVAAVADCFCLA

KDAGFKVVAHMMPDLPNVGVERDLESFREFFENPAFRADGLKIYPTLVIRGTGLYELWKT

GRYRNYPPELLVDIVARILSMVPPWTRVYRVQRDIPMPLVTSGVEKGNLRELALARMEDL

GLKCRDVRTREAGIQDIHHKIRPDEVELVRRDYAANEGWETFLSYEDTQQDILIGLLRLR

KCGRNVTCPELVGRCSIVRELHVYGTAVPVHGRDADKLQHQGYGTLLMEEAERIARKEHR

SKKIAVISGVGTRHYYRKLGYELEGPYMVKCLV*

>OsHAG704

MALKQKGTDAAADPKKRRRVGFSGIDAGVEANECMKVFIARNPDEAGSANSTSLQPFDLN

HFFGEDGKIYGYKNLKINVWISAISFHAYADISFEETSDGGKGITDLKPVLQNIFGENLV

EKDEFLKTFSKECEYLSNVVTDGNVIKHDASIDEDSAVEIVRVELQGAAAFLYCRLVPLI

LLLVEGSTPIDITEHGWEMLLVVKKSAQASSSSNFLVLGFAAVHHFYHYPESTRLRISQI

LVLPPYQGEGHGLRLLETINSISESENIYDVTIEDPSDYLQYIRSSIDCLRLLTFDPIKP

ALCSMVSSLKDTNLSKRTSSLKMVPPSDLAETVRQKLKINKKQFLRCWEILIYLNLDAED

RKSMDNFRACIYDRIKGEILGTSTGPNGKRLVQMPSNFDEETCFAVYWTQDGGDADDQTV

EQQPEDLKTQEQQLNEVVDSQMEEIVEIAKNVTSRGKDKLSVSCSV*

>OsHAM701

MGSMEASTAPENGTAAAAAAAASTACNGAGGGGGAAAASNGGGVERRLRSSAASASWASH

LPLEVGTRVMCRWRDQKLHPVKVIERRKSSTSSSPADYEYYVHYTEFNRRLDEWVKLEQL

DLETVETDVDEKVEDKATSLKMTRHQKRKIDETHVEQGHEELDAASLREHEEFTKVKNIA

KIELGRYEIDTWYFSPFPPEYNDSPKLFFCEFCLNFMKRKEQLQRHMKKCDLKHPPGDEI

YRSGTLSMFEVDGKKNKVYGQNLCYLAKLFLDHKTLYYDVDLFLFYVLCECDDRGCHMVG

YFSKEKHSEESYNLACILTLPPYQRKGYGKFLIAFSYELSKKEGKVGTPERPLSDLGLLS

YRGYWTRVLLEILKKHKSNISIKELSDMTAIKADDILSTLQSLDLIQYRKGQHVICADPK

VLDRHLKAAGRGGLEVDVSKLIWTPYKEQG*
